# Metal–organic frameworks for biomedical applications: bridging materials science and regenerative medicine

**DOI:** 10.1039/d5ra05337d

**Published:** 2025-09-22

**Authors:** Negar Nasri, Marziyeh Azad, Zahra Mehrabi, Ghasem Dini, Afsaneh Marandi

**Affiliations:** a Department of Life Science Engineering, Faculty of Modern Science and Technology, Nano Biotechnology Group, University of Tehran Tehran 1439957131 Iran; b Department of Nanotechnology, Faculty of Chemistry, University of Isfahan Isfahan 81746-73441 Iran g.dini@sci.ui.ac.ir +9837932700 +9837934914; c Department of Biotechnology, Faculty of Biological Science and Technology, University of Isfahan Isfahan 81746-73441 Iran; d Department of Chemistry, Catalysis Division, University of Isfahan Isfahan 81746-73441 Iran

## Abstract

This review explores the biomedical applications of metal–organic frameworks (MOFs), focusing on their potential in bone regeneration. Due to their high porosity, tunable structures, and biocompatibility, MOFs have emerged as promising candidates for bone tissue engineering. This article discusses the synthesis and functionalization of MOFs at the micro- and nanoscale, their interactions with biological environments, and their roles in drug delivery, osteoinduction, and osteoconduction. Recent advancements in theranostic MOF-based scaffolds, which integrate both therapeutic and diagnostic functions, are also highlighted. By mimicking the natural bone architecture, these smart scaffolds promote ossification and angiogenesis while enabling targeted therapy and precision imaging. Despite their great potential, challenges such as metal toxicity, stability, and clinical translation remain. This review emphasizes the transformative role of MOFs in regenerative medicine and discusses strategies to overcome these limitations for future clinical applications.

## Abstract 2

This review explores the biomedical applications of metal–organic frameworks (MOFs), focusing on their potential in bone regeneration.

## Graphical abstract

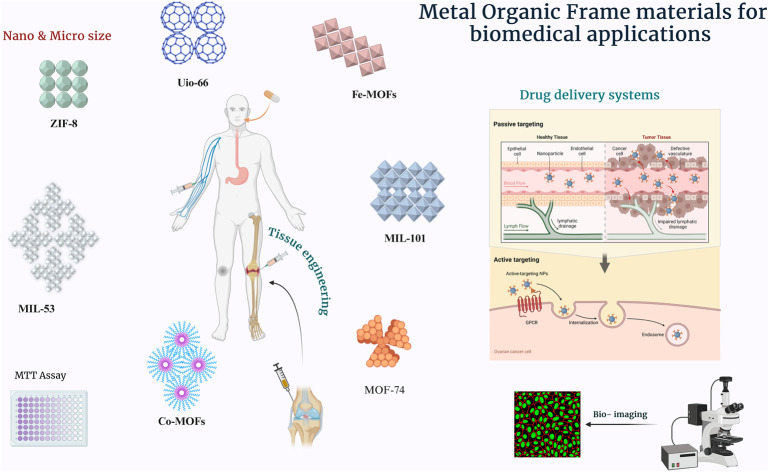

## 1. Introduction

Metal–organic frameworks (MOFs) are highly porous and stable crystalline materials with one-, two-, or three-dimensional architectures, composed of metal ions or clusters coordinated with organic ligands. Since their first introduction in the 1990s, MOFs have undergone significant development, leading to a wide variety of structural topologies and functional classifications.^1^ The vast selection of metal nodes and organic linkers enables the creation of a wide range of MOF structures with diverse functionalities. Based on their structural features and synthetic strategies, MOFs can be broadly categorized into several classes, including isoreticular MOFs (IRMOFs), zeolitic imidazolate frameworks (ZIFs), porous coordination polymers (PCPs), and covalent organic frameworks (COFs), each offering distinct advantages for specific applications.^2^ MOF synthesis employs various self-assembly methods, including hydrothermal, solvothermal, microwave-assisted, sonochemical, and electrochemical techniques.^3,4^ The structural characteristics of MOFs and their derivatives can be tailored through four primary approaches: (1) modifying their metal centers and ligands, (2) controlling their morphology, (3) developing MOF-based hybrid materials, and (4) synthesizing MOF-derived functional compounds. The properties of MOFs, including their composition and structural configuration, play a crucial role in determining their performance across multiple applications. Consequently, achieving precise structural control is essential for optimizing their functionality in various fields.^5,6^

Extensive research has been dedicated to exploring key MOF structures, primarily due to their remarkable characteristics such as high specific surface area (SSA) and well-defined pore uniformity. These structures, with their tunable porosity, provide a versatile platform for scientific investigations.^7^ The unique micro- and nanoscale features of MOFs—including high porosity, large surface area, adjustable pore size, biodegradability, biocompatibility, and adaptability for post-synthesis modifications—make them promising candidates for diverse applications. In recent years, substantial progress has been made in the structural design of MOFs to expand their functional diversity.^8^ Advanced approaches such as multivariate synthesis (introducing multiple linkers or metals into a single framework), post-synthetic modification, and topological control have enabled precise tuning of MOF architectures at the molecular level. The development of hierarchical porous MOFs, flexible and dynamic frameworks, and stimuli-responsive materials has further broadened the scope of their applications, especially in biomedicine, energy storage, and environmental remediation.^9^ Among their most compelling biomedical uses are bone regeneration, targeted drug delivery, and the design of theranostic systems. In recent years, MOFs have played a significant role in the development of integrated diagnostic and therapeutic platforms, owing to their capability to operate both within and outside biological systems.^10,11^

In addition, the biomedical potential of MOFs, particularly in drug delivery and bioimaging, has drawn significant interest from researchers. These structures can encapsulate biomolecules or integrate them during synthesis for various medical applications.^12^ Furthermore, nanoscale and nanostructured MOFs (NMOFs) exhibit extensive surface area, high porosity, and customizable properties, making them ideal for targeted drug delivery and controlled release mechanisms. Despite these advantages, several challenges hinder their clinical translation.^13^ Both the accumulation of MOFs in the body, primarily due to their heavy metal content, and the complexity of regulating drug loading and release remain key concerns. However, extensive research efforts have been directed toward mitigating these issues in recent years. Another crucial factor under discussion is the particle size of MOFs, as it plays a pivotal role in biocompatibility, biodistribution, circulation time, and *in vivo* elimination. Controlling MOF particle size is, therefore, a fundamental aspect of biomedical research.^14^

In recent years, nano- and microscale strategies have gained growing significance in regenerative medicine. Various nano/micromaterials, including particles, composites, and engineered surfaces, have introduced innovative methods for bone tissue regeneration, incorporating a range of advanced techniques.^15^ A comprehensive understanding of natural bone architecture, the diverse materials employed in biomaterial fabrication, and the correlation between bone morphology and functionality is crucial for designing effective bone repair scaffolds. The novelty of this review lies in its integrative perspective: while numerous studies have addressed MOFs in biomedical contexts, a systematic analysis connecting MOF-based nanostructures with the fundamental principles of bone regeneration and the development of intelligent, targeted drug delivery systems has not been previously reported. This review, therefore, highlights how MOF-based nanostructures can be tailored not only for bone repair and regeneration but also for controlled therapeutic release to enhance clinical outcomes.

## 2. Synthesis of MOF structure

MOFs, also known as PCPs, are crystalline, porous materials with infinite lattices. These structures are synthesized from secondary building units, which consist of metal cations or clusters, and polydentate organic ligands that can exhibit various coordination modes. A wide range of metal cations and linkers can be employed, along with potential post-synthesis modifications.^16^ Due to their modular nature, MOFs combine techniques from both organic and inorganic chemistry in their synthesis, allowing the use of diverse building blocks with different properties and functions. This flexibility enables the easy tailoring of specific properties. The diversity of MOFs can be further expanded by increasing the variety of organic ligands, which differ in length, geometry, and functional groups. MOFs can be synthesized in various formats, including linear, square, and triangular structures, each with distinct properties. Divalent, trivalent, and tetravalent metal ions can be incorporated as inorganic units in MOF structures. By carefully selecting appropriate metal ions and organic ligands, it is possible to design MOFs with specific architectural features tailored for advanced applications. The synthesis of MOFs requires energy to break or form bonds, as they contain both organic binders and metal oxides.^17^ The primary goal is to create unique inorganic building blocks while maintaining the integrity of the organic bonds. Various energy-generating devices can be used to synthesize MOFs, and any apparatus capable of producing a controllable amount of heat is suitable for this process. The properties of the resulting MOF depend on the energy source and the synthesis method employed. Several synthesis methods are available for producing MOFs, including hydrothermal, ultrasonic, microwave, mechanochemical, and electrochemical techniques (Table 1). A significant interplay exists between the synthesis method employed for MOFs and their structural classification. Each synthetic approach—solvothermal, microwave-assisted, mechanochemical, electrochemical, or diffusion-based—creates distinct thermodynamic and kinetic environments that determine the crystallization pathway and ultimately the structural features of the resulting MOF.^18^ For instance, solvothermal synthesis, under high-temperature and high-pressure conditions, often favors the formation of thermodynamically stable, highly crystalline 3D frameworks, frequently associated with classical topologies such as face-centered cubic (UiO-66 and MOF-5),^19^ primitive cubic,^20^ or diamond network,^21^ due to the slow self-assembly of well-defined secondary building units (SBUs). Conversely, microwave-assisted and mechanochemical syntheses typically operate under kinetic control, leading to faster nucleation rates, formation of metastable or defect-rich phases, and even lower-dimensional structures (*e.g.*, 1D chains or 2D layers), often classified under less common or distorted topologies. Reaction conditions—including solvent type, temperature, time, pH, and metal-to-ligand ratio—directly influence SBU formation, coordination geometry, and network interpenetration, which are all criteria used in MOF structural classification. Therefore, careful selection and control of synthesis parameters are not only crucial for obtaining desired physical properties but also for targeting specific structural classes and topological families of MOFs.^22^ MOFs have numerous applications, including gas storage, energy conversion, chemical sensing, drug delivery, theranostic nanoplatforms, proton conductivity, and catalysis.^23^ They are also used in industries such as oleochemistry, textiles, transportation, prototype electric vehicles, food packaging, and respiratory systems.^24^

**Table 1:** Various methods used in the synthesis of MOFs

Method	Material	Metal	Ligand	Solvent	Conditions	Ref.
Hydrothermal	Mll-101	Cr (NO_3_)_3_·9H_2_O	H_2_BDC	De-ionized water	180 °C, 5 h	38
	Mn-MOF	MnCl_2_·4H_2_O	H_2_pzca	Water	120 °C, 120 h	39
	Cd/Zr-MOF	Zn^2+^, Cd^2+^	H_2_BDC	DMF	120 °C, 2 h	40
	MOF-5	Zn (NO_3_)_2_·6H_2_O	H_2_BDC	DMF	130 °C, 4 h	41
Ultrasonic	Zn-MOF-U	Zn (CH_3_COO)_2_·2H_2_O	H_3_DTC	Ethanol/water	300 W, 1 h	42
	Cu-MOF	Cu (NO_3_)_2_·5H_2_O	DPA	De-ionized water	100 W, 20 min	43
	Bi-MOF	Bi (NO_2_)_3_·5H_2_O	H_3_BTC	Methanol	600 W, 1 h	44
Microwave	Bi-DBC	Bi (NO_3_)_3_·5H_2_O	H_2_BDC	DMF	400 W, 35 min	45
	PHNiC	(Ni (NO_3_)_2_·6H_2_O)	Trimesic acid	Ethanol/DMF	150 °C, 15 h	46
	UiO-66-GMA	ZrCl_4_	NH_2_-H_2_BDC	DMF, tetrahydrofuran, methacrylate	800 W, 5 to 30 min	47
	MIL-100-Fe	FeCl_3_·6H_2_O	H_3_BTC	H_2_O	130 °C, 10 min	48
Mechanochemical	MOF-74	ZnO	H_4_DHTA	DMF	60 °C, 60 min	49
	MOF-5	Zn(OAC)_2_·2H_2_O	H_2_BDC	DMF/CHCl_3_	423 K, 8 h	50
Electrochemical	Zn-BTC MOF	ZnSO_4_·H_2_O	H_3_BTC	1-Butyl-3-methylimidazolium hexafluorophosphate (IL)	Applied voltage 5 V to 12 V	51
	MOF-199	Cu electrode	H_3_BTC	Electrolyte: tetrabutylammonium tetrafluoroborate (TBATFB)	12 V for 1.5 h	52
	Al-MIL-100	Al(NO_3_)_3_·9H_2_O	H_3_BTC	Electrolyte: KCl	50 mA, 333.15 K	53
	PES/HKUST-1	Cu plate	H_3_BTC	Tetra-*n*-butylammonium perchlorate (TBAP)	2 V, 4 V, 6 V for 6, 12, 24, and 36 h	54

### 2.1. Hydrothermal/solvothermal method

**Fig. 1:**
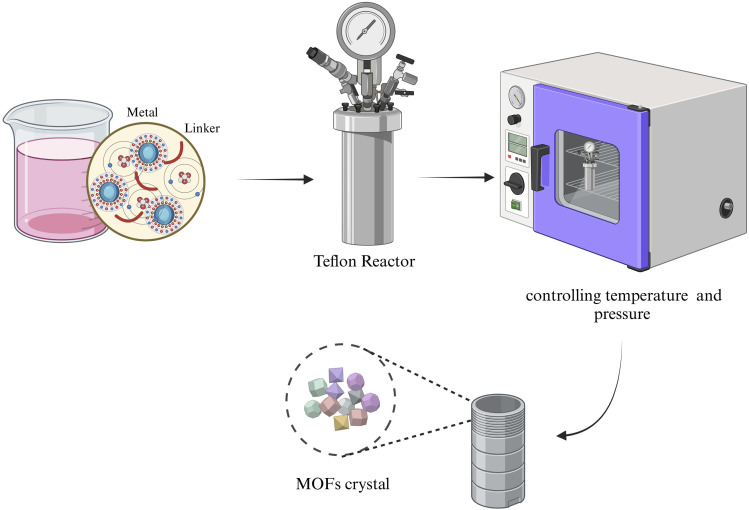
Schematic of the hydrothermal/solvothermal synthesis of MOFs.

The hydrothermal method encompasses various techniques used to crystallize substances from aqueous solutions under high-temperature and pressure conditions.^25^ In the context of solvothermal reactions, chemical processes occur in the presence of a solvent, typically under supercritical or near-supercritical conditions. MOFs are generally synthesized by combining solutions of metal ion salts and organic ligands in solvents, which are then exposed to elevated temperatures (above 100 °C) for a specific duration.^26^ This high-temperature synthesis is employed to achieve optimal crystal yield within a reasonable time frame. During the process, factors such as temperature, pH, and the chemical composition of the reactants are considered key variables. Additionally, surfactants or molecular templates are often used to facilitate the formation of nanoparticles or nanostructures. The pore volume and size of the MOFs can be adjusted by varying the metal ions and organic linkers.^27^ The hydrothermal method has been successful in producing MOFs with diverse sizes, shapes, and crystalline structures. Although thousands of MOFs have been synthesized, several factors—such as size, surface charge, shape, stability, and toxicity—must be carefully considered before their use in biomedical applications. These parameters are interdependent, often influencing one another.^28^Fig. 1 presents the steps involved in a typical synthesis process using both hydrothermal and solvothermal methods.^29^

### 2.2. Mechanochemistry

**Fig. 2:**
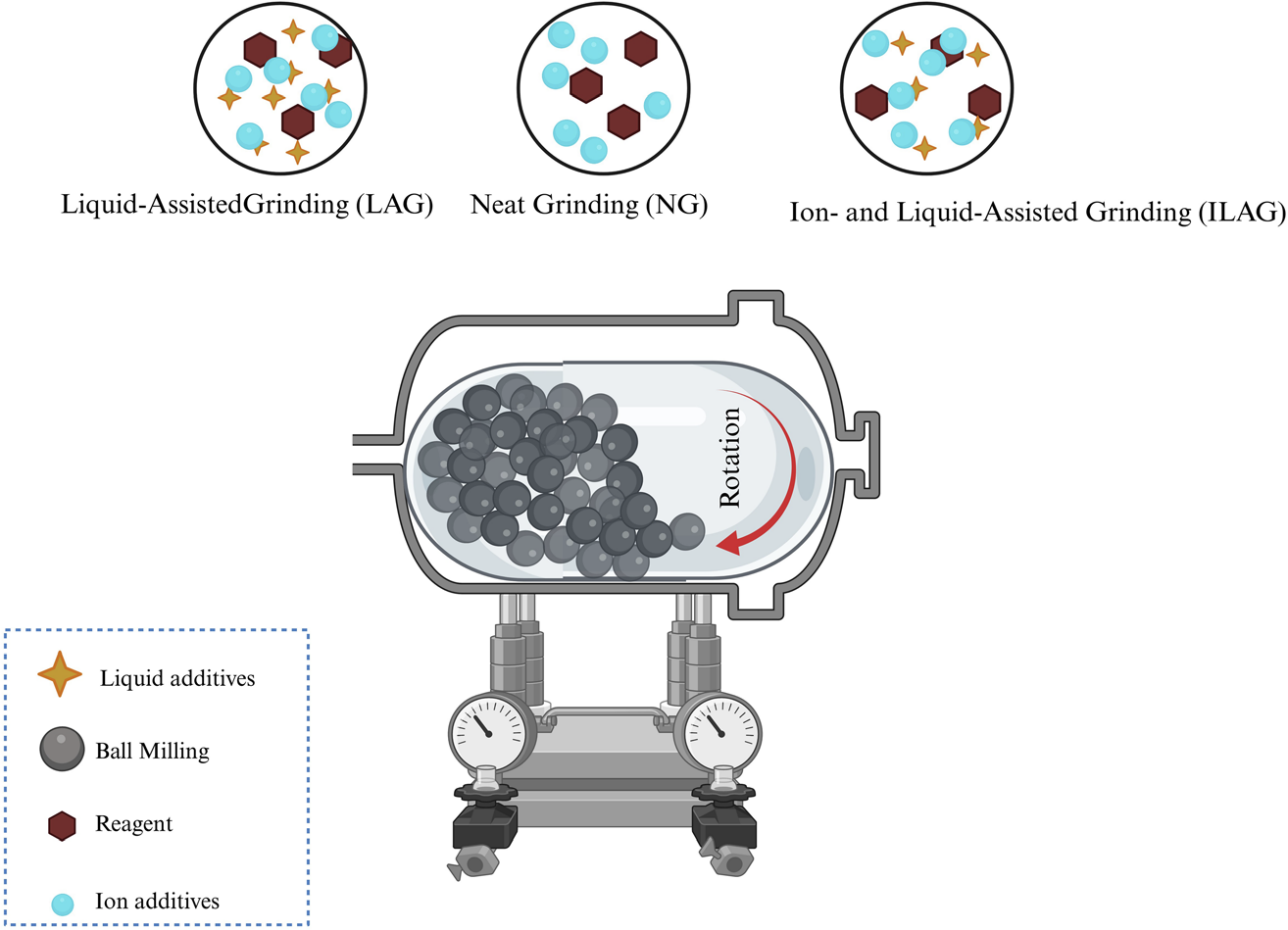
Schematic illustration of mechanochemical methods.

Mechanochemical reactions involve the direct absorption of mechanical energy by reactants, usually solids, during the milling process. In ball milling, chemical reactions are triggered by the friction and impact between the balls and reactants (Fig. 2).^30^ For a reaction to occur, the balls must strike the ground with sufficient force to break chemical bonds; otherwise, only elastic deformation will happen. The high-energy grinding generates structural stress, leading to bond rupture and the formation of reactive radicals. This exposes reactive atomic layers at the interface of the solid reactants, thereby promoting chemical reactions. Mechanochemical synthesis typically involves grinding solid precursors in a ball mill without the use of solvents, enabling the use of metal sources that are insoluble in conventional solvents for MOF synthesis. For instance, metal oxides, which are insoluble in common solvents, can be employed in place of salts, offering a safer and more environmentally friendly option. Another mechanochemical approach is Liquid-Assisted Grinding (LAG), which uses a solvent to increase the mobility of metal ions and organic bonds, facilitating the formation of coordination bonds.^31^ A variation of this, Ion and Liquid-Assisted Grinding (ILAG), incorporates both salt and liquid into the milling process. These additives not only help dissolve solid reagents but also improve the reactivity of the substrate, thus enhancing the efficiency of milling by creating a more homogeneous reaction mixture.^30–32^ It is crucial to carry out MOF synthesis with a focus on ecological and economic considerations. Therefore, the use of inexpensive and readily available precursors, such as metal oxides, is especially appealing. Taheri *et al.*^33^ achieved the first dry conversion of ZnO into Zeolitic Imidazolate Framework-8 (ZIF-8) by grinding nanosized ZnO and 2-methylimidazole (HmIm) for 96 hours, resulting in a porous material with a SSA of 1480 m^2^ g^−1^, which is higher than the typical SSA of around 1200 m^2^ g^−1^ for this type of MOF.

### 2.3. Ultrasound and microwave methods

Ultrasound irradiation is a non-traditional technique that has gained significant attention due to its advantages, including ease of use under ambient conditions, straightforward operation, selective product formation, and reduced reaction time. Over recent years, the application of ultrasound in chemical reactions has expanded, leading to substantial experimental advancements. In this method, powerful ultrasound waves (ranging from 20 kHz to 1 MHz) are applied to drive chemical reactions. In liquids, sonochemical reactions are driven by the formation, growth, and collapse of bubbles, a phenomenon known as acoustic cavitation. This process generates intense localized heating, high pressures, and brief lifetimes of the bubbles, which significantly influence the reaction environment.^34^ Recent studies have shown that MOF-based porous materials can be efficiently synthesized, purified, and modified using ultrasound techniques. Ultrasound has proven to be an essential tool in MOF research, enabling the production of smaller particle sizes under milder conditions compared to traditional methods that require high temperatures and extended reaction times.^35^

In the microwave method, the reaction mixture is heated using microwave radiation, which accelerates the crystallization of MOFs compared to traditional heating methods. Microwaves promote the rotation of specific molecules within the mixture, increasing the chances of molecular collisions and the production of heat. This, in turn, facilitates the reaction process. Microwave-assisted synthesis also offers several advantages over hydrothermal synthesis, such as rapid heating, faster reaction kinetics, phase purity, higher yields, and improved reliability and reproducibility.^36^

### 2.4. Electrochemical methods

The electrochemical method is a continuous reaction process for synthesizing MOFs driven by redox reactions. In this technique, metal ions act as the anode, while the linker molecule is dissolved in the reaction vessel along with the electrolyte. When protic solvents are present, metal ions from the anode do not deposit on the cathode. This process offers several advantages over traditional solvothermal or microwave-assisted methods, such as milder reaction conditions, easier crystallization, and shorter reaction times. Furthermore, electrochemical synthesis enables the continuous production of MOFs. The final structure of the MOFs is significantly influenced by factors like voltage, current density, solution composition, electrolyte properties, and solvent selection.^37^

## 3. MOF structure

Classifying MOFs is crucial for understanding the relationship between their structure and properties, identifying new structures with desirable characteristics, and designing MOFs for specific applications. Such classifications also play a key role in optimizing certain applications. A wide variety of applications have been explored, but no one system can adequately describe the structural properties of MOFs. An overview of common structural classifications can be found below, which provides insight into the arrangement of metal nodes, organic linkers, and void spaces within MOFs.^55^

### 3.1. Topological classification

**Fig. 3:**
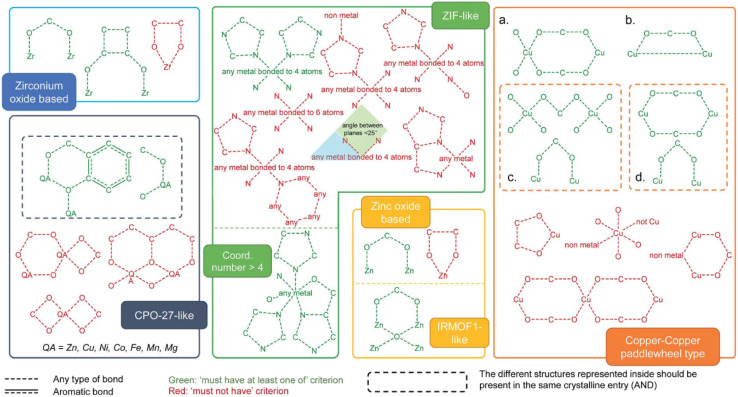
A schematic depiction of various MOF families, including structures such as zirconium oxide, MOF-74/CPO-27-like frameworks, ZIF-like structures, zinc oxide, IRMOF-like materials, and Cu–Cu paddle-wheel-based frameworks^58^ (reproduced from Moghadam P. Z., Li A., Liu X. W., Bueno-Perez R., Wang S. D., Wiggin S. B., Wood P. A., and Fairen-Jimenez D., *Chem. Sci.*, 2020, **11**, 8373–8387 under a Creative Commons Attribution 3.0 Unported Licence). (a–d) Diagrams used to look for structures containing Cu–Cu paddlewheels. The dotted box for (c) and (d) means the structures inside should be considered as one single query. The red diagrams are queries used to eliminate undesired structures.

Topological classification is one of the most fundamental and widely adopted approaches to categorizing MOFs, as it provides an abstract and geometry-based perspective on framework structure (Fig. 3). This method involves reducing the MOF structure to a simplified network or net, where metal clusters or SBUs are represented as nodes, and organic linkers as edges, allowing for comparison of diverse frameworks based on their underlying connectivity rather than chemical composition. Through this lens, MOFs are grouped into families with distinct topologies, such as fcu, pcu, dia, and others, each with unique geometric and structural characteristics. While topological analysis offers a powerful framework for understanding the spatial organization of MOFs, it is often complemented by other classification schemes that highlight different aspects of MOF architecture.^56^ For example, classification based on dimensionality focuses on the extension of connectivity in 1D, 2D, or 3D networks; cage-based classification emphasizes the presence and geometry of discrete polyhedral cavities; functional group-based classification considers the chemical nature and distribution of active sites within the framework; and supramolecular classification examines higher-order organization and host–guest interactions. These complementary approaches provide a more comprehensive understanding of structure–property relationships in MOFs and guide the rational design of materials for specific applications.^57^

### 3.2. Classification based on dimensionality

MOFs can also be categorized according to their structural dimensionality, which defines the number of spatial dimensions the framework extends across. These materials are generally classified into three main types: zero-dimensional (0D), one-dimensional (1D), and three-dimensional (3D). In 0D MOFs, discrete clusters or individual metal centers are present, whereas 1D MOFs exhibit chain-like configurations. In contrast, 3D MOFs develop extensive networks with porous architectures. The classification provides essential insights into the connections and spatial arrangements of metal nodes and organic linkers in MOFs.^58^

### 3.3. Cage-based classification

Certain MOFs contain substantial voids or internal cavities, and these materials are categorized based on the geometry, dimensions, and interconnectivity of these empty spaces. For example, ZIFs commonly exhibit cubic or octahedral enclosures. Conversely, structures such as metal–organic polyhedra (MOPs) and coordination polymers (CPs) frequently display hexagonal prismatic or concave coordination cages. This classification emphasizes the distinctive architectural characteristics of MOFs and their potential for various applications, which are influenced by the nature of these precisely structured voids.^55^

### 3.4. Functional group-based classification

**Fig. 4:**
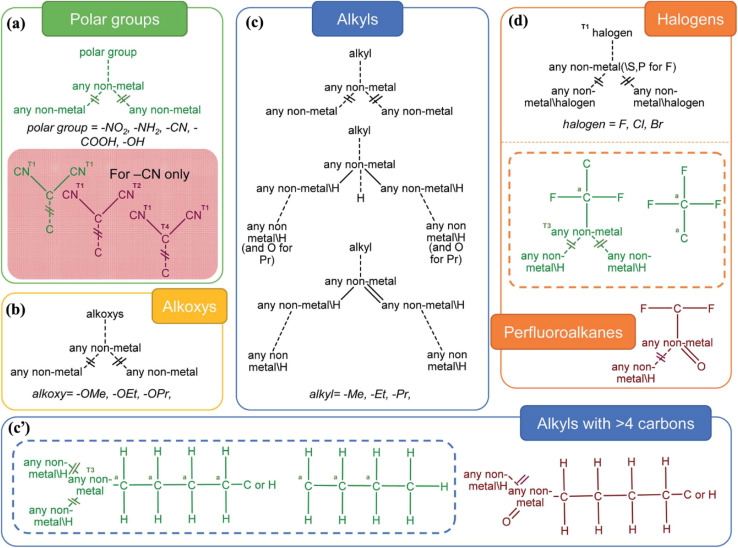
Selection rules established for identifying MOFs that share common functional groups within the CSD MOF subset. (a) Polar functionalities (–NH_2_, –NO_2_, –CN, –COOH, and –OH). In the case of –CN, the red box highlights search queries focused on dicyanides, which were intentionally excluded. This exclusion was performed by combining one “required” query with two “prohibited” queries. As a result, the green diagram represents an overall negative case, while the red diagrams illustrate double-negative conditions; (b) alkoxy groups (methoxy, ethoxy, propyloxy); (c) alkyl chains (methyl, ethyl, propyl); (c′) longer alkyl chains with more than four carbons on the left; and (d) halogens (–F, –Cl, –Br) together with structures containing perfluoroalkane substituents^58^ (reproduced from Moghadam P. Z., Li A., Liu X. W., Bueno-Perez R., Wang S. D., Wiggin S. B., Wood P. A. and Fairen-Jimenez D., *Chem. Sci.*, 2020, **11**, 8373–8387 under a Creative Commons Attribution 3.0 Unported Licence).

Another approach to categorizing MOFs involves examining the functional groups present in their organic linkers (Fig. 4). These linkers can incorporate a diverse range of functional moieties, including polar groups (–NH_2_, –NO_2_, –CN, –COOH, –OH), alkoxy groups (such as methoxy, ethoxy, and propyloxy), alkyl chains (*e.g.*, methyl, ethyl, propyl, and longer hydrocarbons), and halogens (–F, –Cl, –Br). The presence of these groups significantly affects the chemical behavior of MOFs, altering their reactivity, selectivity, and adsorption characteristics. This classification underscores the chemical versatility of MOFs and their application potential, which is largely determined by the nature of the functional groups within their structure.^58^

### 3.5. Supramolecular classification

MOFs are classified based on their non-covalent interactions and self-assembly patterns in their structures, according to the supramolecular approach. This method takes into account key factors such as hydrogen bonding, π–π interactions, host–guest dynamics, and coordination forces between metal centers and guest species. MOF stability, porosity, and overall functionality are significantly influenced by non-covalent forces.^59^ By classifying MOFs according to these interactions, scientists can refine their molecular design and better control their assembly processes. It is essential to recognize that classification schemes for MOFs are not strictly independent, and multiple methods can be integrated to develop a more holistic understanding of their structures. Advances in computational methodologies—including graph theory, machine learning, and data mining—have enabled the creation of automated classification algorithms, enhancing the structural analysis and predictive capabilities of MOFs.^60^ Nevertheless, due to their intricate architectures and immense structural diversity, no single universal classification framework has yet been established.^55^

## 4. Biological behaviors underlying MOF-nanocomposite scaffolds

As MOFs are being investigated for use in medicine, the field has made significant progress, yet many aspects remain to be thoroughly examined, including the essential need to assess their toxicity. Despite the extensive study of nearly ninety thousand MOFs, evaluating and refining their physical and chemical properties within relevant biological systems for potential medical applications is crucial. Based on current knowledge, concerns about the safety of MOFs in biological systems remain valid. It is widely recognized that materials designed for contact with the human body must satisfy strict requirements before use. Beyond fulfilling a specific purpose (with a suitably chosen structure and properties), they must also be evaluated for their durability, degradability, and ability to coexist with living tissue.^55,61^ The biocompatibility of metal components and bridging ligands is crucial in the design of MOF scaffolds. Certain metals, such as chromium, exhibit toxicity, whereas others, including zinc, copper, iron, and manganese, are vital for biological functions. For example, iron is a key element of hemoglobin, with its concentration in blood plasma averaging around 128.4 ± 18.1 g L^−1^.^62^ Tissues also contain various metals like Co (68 mM), Mn (180 mM), Ni (2 mM), and Zn (180 mM).^63^ Prominent metals for biomedical engineering applications include Ca, Mg, Zn, Fe, Ti, Mn, Zr, and Co ions, all of which have a lethal dose 50 (LD_50_) of less than 25 g kg^−1^.^64^ Factors such as metal concentration, oxidation state, and toxicity can further influence the biocompatibility of MOFs. Another important consideration affecting scaffold biocompatibility is the use of organic linkers, which should be non-toxic and easily removable under physiological conditions. Organic linkers such as polycarboxylic and imidazolate linkers, due to their high polarity, are generally not very toxic and can be easily eliminated under physiological conditions.^62^ Additionally, it has been discovered that besides chemical characteristics, factors like particle size, shape, and aggregation play a crucial role in determining the biocompatibility of MOFs. Therefore, carefully designing and adjusting these factors can help reduce the inherent harmfulness of these structures, making them more suitable for use in biomedical applications. Strategies such as green chemistry and surface modifications have been identified as effective approaches to minimize MOF toxicity.^55^

### 4.1. Green MOFs for medical applications

Wiśniewska *et al.*,^55^ in their study, emphasized the potential of modifying MOF chemistry using environmentally friendly ligands, linkers, and solvents as a means to reduce toxicity. They acknowledged the challenges involved, as eco-friendly alternatives often result in decreased performance. However, the potential benefits for medical applications are substantial. Recent studies have focused on creating, synthesizing, and assessing the efficacy of environmentally friendly MOFs for medical purposes. Grape *et al.*^65^ demonstrated in one study that renewable, plant-based linkers and solvents can yield a completely biocompatible and environmentally friendly MOF (SU-101) with very low cytotoxicity and good colloidal stability. Another study by Grape *et al.*^65^ confirmed that MOFs containing cyclodextrin (a semi-natural product) as the organic linker exhibited no toxicity in cell lines, demonstrating good drug loading and controlled release capabilities. Agostoni *et al.*^37^ compared a green, HF-free synthesis route for MIL-100(Fe) MOF to the conventional method, finding that the green MOFs had better drug encapsulation efficiency and were equally biocompatible while also improving the yield and synthesis time. However, the study by Jiang *et al.*^66^ indicated that bio-derived MOFs do not necessarily guarantee complete biocompatibility, as Bio-MOF-1 showed some *in vitro* and *in vivo* toxicity at higher concentrations.

The use of green components in the synthesis of MOFs can contribute to increased biological safety, but does not necessarily ensure complete biocompatibility, as toxicity is influenced by many factors. Therefore, further research is needed to confirm the superiority of green MOFs over conventional MOFs in terms of non-toxicity and their utility for biomedical applications.

### 4.2. Surface modification of MOFs for biosafety

Besides using green precursors and solvents, modifying the surface of MOFs is crucial in reducing their toxicity, as surface properties control interactions with the biological environment. Proper surface modifications can limit direct contact between the MOF and cell surfaces. Research has shown that several modifications enhance MOF biosafety, including coatings with biomolecules and covalent bonding. For instance, lipid coatings have proven effective in improving the biocompatibility of MOFs. Wuttke *et al.*^13^ found that lipid bilayers around MIL-100(Fe) and MIL-101(Cr) nanoparticles enhanced their biocompatibility, although toxicity remained at higher doses (100 μg mL^−1^). Similarly, Ploetz *et al.*^67^ reported reduced HeLa cell viability when exposed to lipid-coated MIL-100, while bare MOFs exhibited less toxicity due to lower internalization rates. Lipid functionalization also enhances MOF stability in physiological conditions and boosts uptake efficiency for drug delivery applications, as observed by Yang *et al.* with PCN-223.^68^

Other biomaterials, such as chitosan and heparin, have also been used to modify MOFs, showing improved stability and reduced immune responses. For example, chitosan-coated MIL-100 demonstrated reduced inflammatory cytokine production in immune cells, while both coated and uncoated MIL-100 showed minimal toxicity to colorectal carcinoma cells.^69^ Moreover, modifications with molecules like poly(ethylene glycol) (PEG) and hyaluronic acid have enhanced MOF hydrophilicity, drug release, and cellular uptake. PEG-coated Zr-fum MOFs, for example, exhibited selective cytotoxicity toward cancer cells while being well-tolerated by healthy cells.^70^ Similarly, hyaluronic acid-modified ZIF-8 improved MOF biocompatibility and efficacy in photodynamic therapy.^71^ Overall, surface modifications hold significant potential to overcome MOF toxicity and enhance their applicability in drug delivery and biomedical fields. Further studies on bio-based modifications, such as with peptides or nucleic acids, are necessary to continue improving MOF performance.

## 5. MOF fabrication for medical applications

Therapeutic agents often face challenges such as low bioavailability, high rates of side effects, and quick elimination from the body. Nanoparticle-based systems offer a promising solution to these issues due to their small size, large surface area, enhanced drug loading capacity, and improved pharmacokinetics.^72^ The small dimensions and high sensitivity of nanoparticles make them particularly effective in drug delivery systems (DDSs). Additionally, nanoparticles excel in active, smart, and targeted drug delivery applications.^73^

Various nanomaterials offer a range of benefits and drawbacks concerning their toxicity, stability, drug-loading capacity, and other key properties. NMOFs are a compelling class of crystalline porous materials, constructed from a variety of metal ions and organic linkers.^74^ MOFs have a broad range of potential applications, including gas storage, catalysis, magnetism, sensing, and separation, owing to their high porosity, large surface areas, biodegradability, biocompatibility, and the ability for post-synthetic modifications. Increasing interest in their biomedical applications has emerged, particularly in drug delivery and bioimaging.^75^ Biomolecules can be encapsulated or incorporated into MOFs for specific biomedical purposes, as outlined in Table 2. Due to their customizable features, NMOFs serve as excellent carriers for targeting specific sites in the body, facilitating controlled drug release. Recent advancements in NMOFs have demonstrated their potential in cancer-targeted therapies.^60^ Moreover, functionalization of the inner and outer surfaces of MOFs has enabled the development of reliable systems for diagnostic and therapeutic use.^74^

**Table 2:** MOFs in biomedical applications

MOF	Cargo	*In vitro*/*in vivo*	Application	Ref.
ZIF-8 MOF	Bioactive glass	MG-63 cell line (*in vitro*)	Osteogenic properties/antimicrobial properties	76
CuO@ZnO MOF coated on a titanium implant	Titanium implant	Human bone marrow mesenchymal stem cells (hBMSCs)	Osteoinductive properties/angiogenesis ability/antibacterial properties	77
Mg-MOF-74	Mesoporous silica shell	BMSCs cell	Cell proliferation and bone regeneration properties	78
Mg-MOF-74	Co-drug delivery ibuprofen–curcumin	—	Supplement drug administration *via* orally ingested tablets	79
Uio-67 MOF	5-FU	A549 and HeLa cell lines	Antitumor efficacy, biocompatibility, and simultaneous release of hydrophobic and hydrophilic drugs at the tumor site	80
ZIF-90 coated on chitosan	Methotrexate	HepG2 cells, prostate cancer DU145 cells and gastric cancer SGC7901 cells	High MTX drug loading, cancer-targeted release, and good biocompatibility	81

### 5.1. Drug delivery application of NMOFs

Effective drug release control and enhanced drug efficacy can be achieved through the use of a well-designed and efficient DDS (Table 3). The functionality of MOFs can be enhanced through surface modifications. With their large pore sizes and surface areas, MOFs are ideal candidates for drug encapsulation, offering flexible post-synthetic grafting options for attaching therapeutic molecules, as well as inherent biodegradability. These attributes make MOFs highly promising as drug carriers in therapeutic applications. The primary objective is to deliver drugs to specific sites in the body. Additionally, they play a significant role in regulating drug release and enhancing cellular uptake. These features make MOFs one of the most advanced DDS options.^82,83^ BioMOFs, where the organic linker is entirely biological, are often designated as Bio-MOF-xxx, where “xxx” refers to a specific identifier assigned upon their synthesis and registration. In contrast, a BioMOF may also refer to a biocompatible MOF capable of acting as a carrier for biological molecules or drugs.^84^ MOF-based nanocarriers can generally load drugs in two distinct ways, owing to their high porosity and surface area.

**Table 3:** Comparison of drug delivery performance of different MOF systems

MOF type	Loaded drug	Drug loading capacity (wt%)	Release mechanism	Target	Ref.
MIL-101(Fe)	Metronidazole	10%	The drug release is controlled by the gradual degradation of the MOF in the presence of phosphates and the desorption of the drug from the MOF surface	Antibacterial	102
Bi-MIL-88B	5-Fluorouracil	29.8%	PH	Tumor	103
Br-MOF-5	Oridonin	39.97%	PH	Passive targeting	104
Mof-199	Doxorubicin	39.58%	PH, GSH, NIR	Tumor	105
		73.26%			
ZIF-90	10-Hydroxycamptothecin	22.3%	PH, ATP	Tumor	106
Zr-MOF	5-Fluorouracil	56.5%	PH	Tumor	107

Due to their active surface, NMOFs can adsorb active molecules onto their surface through a process known as surface adsorption. In this method, pre-synthesized MOFs are typically agitated in a solution containing drug molecules to facilitate adsorption. The primary forces driving this process include hydrogen bonding, π–π interactions, and van der Waals forces.^85^ The second method takes advantage of the high porosity of MOFs, with pore sizes tunable from microporous to mesoporous, allowing various functional molecules to be accommodated within the pores.^86^ MOFs provide a protective environment from external factors as well as prevent the leaching of the loaded substrates. Pore encapsulation *via de novo* synthesis is a highly efficient approach for incorporating functional molecules into MOFs. In this process, MOF formation and substrate encapsulation take place simultaneously. This allows the immobilization of larger molecules, even those exceeding the pore size of the MOFs, within the MOF cavities. However, the substrate must remain stable under the synthetic conditions. This method has been widely employed for encapsulating anticancer drugs within MOFs for intracellular delivery and controlled release.^87^ Researchers have increasingly employed pillar[*n*]arenes to enhance the precision, responsiveness, and performance of MOF-based drug delivery systems. Pillar[*n*]arenes are synthetic macrocyclic compounds composed of *para*-linked 1,4-dialkoxybenzene units arranged in a rigid cylindrical shape, forming a well-defined internal cavity. These molecules can form reversible host–guest complexes, respond to environmental stimuli such as pH, temperature, or specific ions, and be chemically modified at both rims to tune their interactions. They are also relatively biocompatible, which makes them suitable for biomedical applications. When combined with MOFs, pillar[*n*]arenes can act as molecular gates, temporarily sealing the pores of the MOF to prevent premature drug leakage. Exposure to specific triggers, such as the acidic tumor microenvironment or certain chemical agents, causes the gates to open, allowing controlled and site-specific drug release. This design integrates the high loading capacity and tunable porosity of MOFs with the responsive behavior of pillar[*n*]arenes. Studies have demonstrated that such hybrid systems can improve therapeutic efficacy while reducing side effects.^88^

In a notable study by Tan *et al.*^89^ (2015), a smart, stimuli-responsive drug delivery system was developed using MIL-101, a chromium-based metal–organic framework (MOF) known for its high surface area and excellent thermal and chemical stability. The researchers engineered the surface of MIL-101 nanoparticles with pillar[5]arene-based supramolecular gates, leveraging their well-defined host–guest chemistry. The gating mechanism relied on the formation of reversible complexes between pillar[5]arene and alkyl diammonium salts. These complexes acted as molecular switches, effectively blocking the pore openings of the MOF in the “closed” state to trap guest molecules like Nile Red. Upon exposure to external chemical stimuli—such as solvent polarity changes (*e.g.*, DMSO) or variations in pH—the host–guest interactions were disrupted, causing the gates to “open” and release the encapsulated cargo. This design showcases an elegant integration of porous materials and supramolecular chemistry, resulting in a chemically gated MOF platform capable of controlled and reversible molecular release. The system not only highlights the potential of MOFs in smart delivery applications but also offers a modular strategy for constructing responsive nanocarriers tuned by specific external cues.

**Fig. 5:**
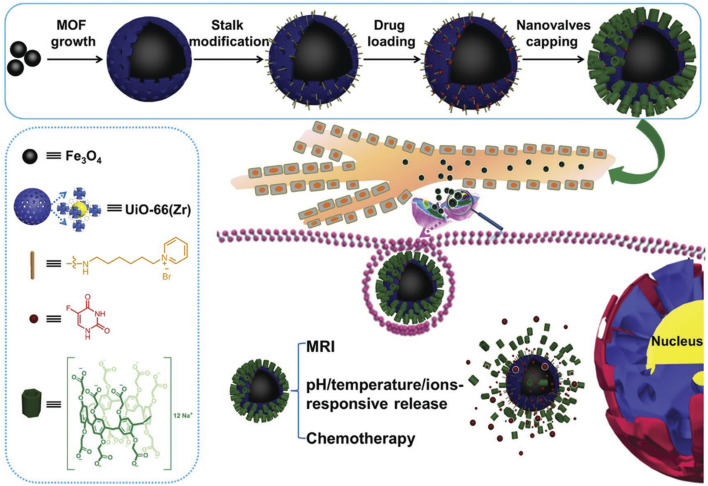
A schematic representation of the synthesis steps and functional mechanism of the Fe_3_O_4_@UiO-66@WP6 theranostic nanoplatform, along with the structural details of its key components^90^ (reproduced with permission from Wu M. X., Gao J., Wang F., Yang J., Song N., Jin X., Mi P., Tian J., Luo J., Liang F. and Yang, Y. W., *Small*, 2018, **14**(17), p. 1704440. Copyright 2018 Wiley-VCH Verlag GmbH & Co. KGaA).

In an innovative study, Wu *et al.*^90^ developed a multistimuli-responsive core–shell hybrid nanoplatform combining Fe_3_O_4_ magnetic nanoparticles with a shell of UiO-66 metal–organic framework (MOF), further functionalized with pillar[6]arene-based supramolecular nanovalves. UiO-66, a Zr-based MOF, was selected due to its exceptional chemical and thermal stability, biocompatibility, and large surface area suitable for drug encapsulation. This Fe_3_O_4_@UiO-66 construct effectively merges structural robustness with functionality, forming a responsive and highly adaptable system for drug delivery applications (Fig. 5). The platform operates *via* multiple external stimuli, including pH changes, elevated glucose levels, temperature variations, and magnetic fields. The Fe_3_O_4_ core enables magnetic targeting and imaging *via* MRI, while the porous UiO-66 shell stores therapeutic cargo. The outer layer is equipped with pillar[6]arene nanovalves, which serve as molecular gates—blocking the MOF pores through reversible host–guest interactions. These interactions are sensitive to environmental cues; for instance, in the acidic and glucose-rich tumor microenvironment, the gates dissociate, resulting in triggered and site-specific drug release. This multidimensional design allows for precise spatiotemporal control of drug delivery, minimal off-target effects, and potential for combined therapeutic and diagnostic (theranostic) applications. The study exemplifies how integrating stable MOF structures like UiO-66 with smart supramolecular elements like pillararenes can produce advanced, responsive drug delivery systems tailored to the complexity of biological environments—especially in cancer treatment.

**Fig. 6:**
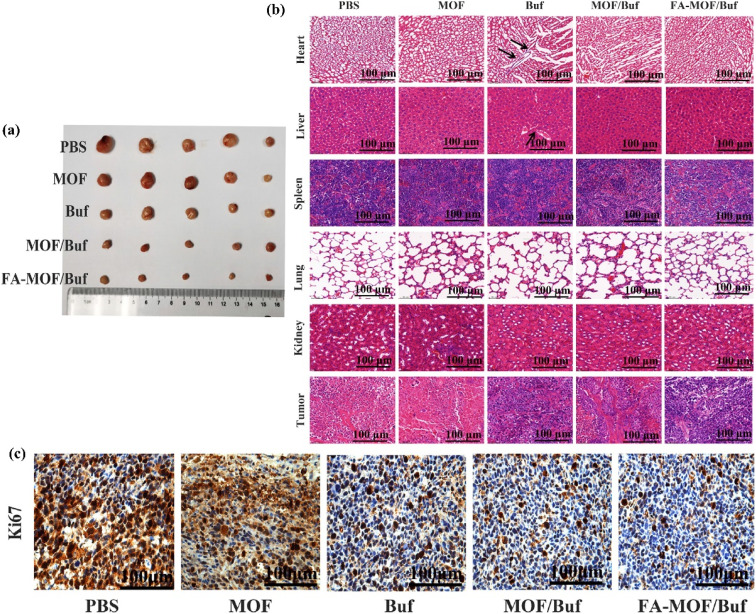
(a) Representative tumor tissues excised from mice at the end of treatment, (b) histopathological examination of major organs (heart, liver, spleen, lungs, and kidneys) and tumor tissues after treatment on the xenografted mice, and (c) expression of Ki67 in cancer tissues, measured by IHC staining^91^ (reproduced from Zeng H., Xia C., Zhao B., Zhu M., Zhang H., Zhang D., Rui X., Li H. and Yuan Y., *Front. Pharmacol.*, 2022, **12**, p. 747992 under a Creative Commons Attribution 4.0 International License).

Zeng *et al.*^91^ developed pH- and redox-responsive MOFs functionalized with folic acid (FA-MOF/Buf) for targeted delivery of Bufalin (Buf) to cancer cells through receptor-mediated endocytosis, thereby significantly enhancing delivery accuracy. Their findings suggested that FA-MOF could serve as a promising nanocarrier for Buf, improving its anticancer efficacy. Upon administration of Buf at a dose of 2 mg kg^−1^, tumor progression was notably suppressed compared to the control group (Fig. 6a). Furthermore, all nanoparticle-based Buf formulations exhibited greater tumor inhibition than free Buf, likely due to enhanced accumulation of larger particles within tumors *via* the enhanced permeability and retention (EPR) effect. This enhanced therapeutic performance was also observed in *in vitro* cytotoxicity assays, further supporting the improved anticancer potential. Among the groups tested, the FA-MOF/Buf formulation displayed the most substantial tumor suppression, attributed to the active targeting ability provided by folic acid modification and the macromolecular EPR effect. Immunohistochemical (IHC) analysis of Ki67 expression in tumor tissues revealed lower levels of positive staining in the groups treated with free Buf, MOF/Buf, and FA-MOF/Buf compared to the MOF and PBS controls. The FA-MOF/Buf group showed the lowest Ki67 expression, followed by MOF/Buf and then free Buf (Fig. 6c), establishing the order of antitumor effectiveness as FA-MOF/Buf > MOF/Buf > MOF ≈ PBS. Histopathological examination of major organs (heart, liver, spleen, lungs, and kidneys) and tumor sections stained with H&E indicated signs of tissue damage in the heart and liver of the free Buf group, including cardiomyocyte necrosis and increased connective tissue formation. In contrast, no significant pathological changes were observed in the nanoparticle-treated or control groups. Additionally, treatment groups demonstrated larger necrotic and apoptotic regions within tumor tissues compared to controls, with the most pronounced damage evident in the FA-MOF/Buf group (Fig. 6b).

**Fig. 7:**
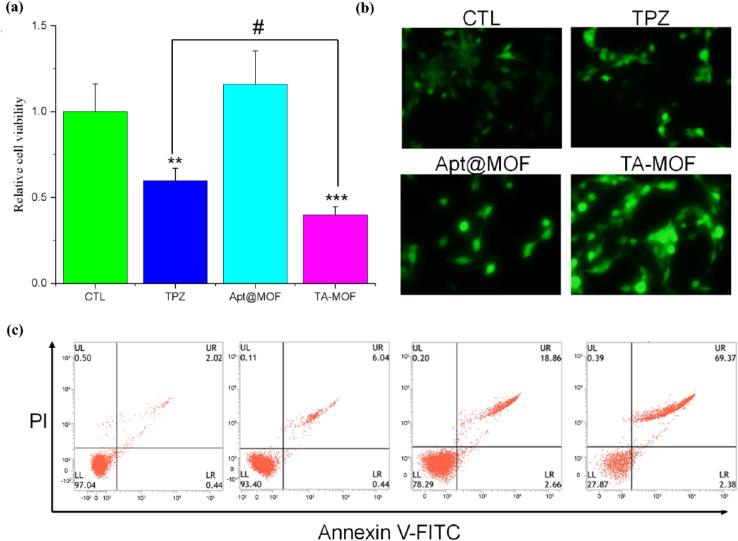
The antitumor effect of TA-MOF *in vitro*: (a) CCK8 detected the changes in cell viability after the TA-MOF treatment. The data are presented as the mean ± SEM (*n* = 3; **p < 0.01 *versus* the control group, ***p < 0.001 *versus* the control group, #*p* < 0.05 *versus* the indicated group), (b) apoptosis was detected by flow cytometry after the TA-MOF treatment, and (c) ROS production was observed by fluorescence imaging and DCFH-DA staining^92^ (reproduced from Wang X., Chen Q. and Lu C., *Molecules*, 2022, **27**(13), p. 4247 under a Creative Commons Attribution 4.0 International License).

In a recent study, Wang *et al.*^92^ (2022) developed a targeted drug delivery system by incorporating aptamer-based recognition units into an MOF. The system, named TPZ@Apt-MOF (TA-MOF), employs an iron-based MOF as the carrier, which is surface-modified with the AS1411 aptamer to actively target nucleolin-overexpressing tumor cells. Inside the MOF, the hypoxia-activated prodrug tirapazamine (TPZ) is encapsulated. Once the nanocarrier reaches the tumor microenvironment, it undergoes degradation, releasing ferrous ions (Fe^2+^) and TPZ. The Fe^2+^ ions initiate a Fenton reaction, generating reactive oxygen species (ROS) that contribute to chemodynamic therapy. This process also depletes intracellular oxygen and glutathione, which intensifies hypoxia and enhances the tumor's sensitivity to TPZ. Consequently, this system represents a highly selective, stimuli-responsive platform well-suited for precision oncology applications. To assess the anticancer efficacy of TA-MOF, 4T1 mammary carcinoma cells were treated and evaluated for changes in viability. As illustrated in Fig. 7a, the TA-MOF treatment group exhibited a markedly greater reduction in cell viability compared to both the free TPZ and MOF groups. Concurrently, apoptosis levels were measured (Fig. 7b), revealing significantly higher rates of programmed cell death in the TA-MOF group than in the control treatments. This enhanced effect is believed to be linked to the generation of ferrous ions (Fe^2+^) during the degradation of MOF structures, which may potentiate the therapeutic action of TPZ. To investigate this mechanism, intracellular ROS levels were analyzed (Fig. 7c). Cells treated with TA-MOF displayed a substantial increase in ROS production, as indicated by noticeably stronger fluorescence signals compared to those treated with free TPZ or MOF alone. Overall, TA-MOF enhances ROS generation through the combined effects of the Fenton reaction and hypoxia-activated prodrugs, thereby effectively inducing apoptosis in tumor cells.

**Fig. 8:**
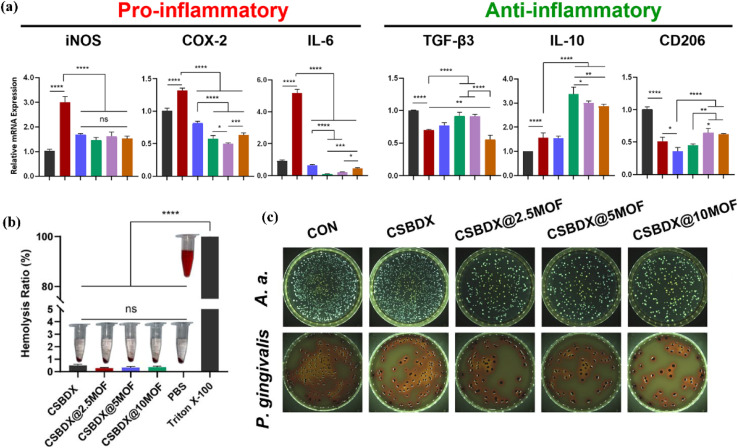
(a) The antioxidant and immunomodulatory properties of CSBDX@MOF. The quantitative analysis of DPPH E and macrophage polarization-related protein (iNOS and CD206) expressions, (b) the biocompatibility of CSBDX@MOF: hemolysis ratio of rabbit erythrocytes incubated with hydrogels, and (c) antibacterial effect: representative images of colony formation assay and bacterial survival histogram (*n* = 4, *: *P* < 0.05, ***: *P* < 0.001, ****: *P* < 0.0001, ns no significance)^93^ (reproduced from Luo Q., Yang Y., Ho C., Li Z., Chiu W., Li A., Dai Y., Li W. and Zhang X., *J. Nanobiotechnol.*, 2024, **22**(1), p. 287 under a Creative Commons Attribution 4.0 International License).

Luo *et al.*^93^ engineered an innovative injectable self-healing hydrogel that integrates magnesium–gallic acid metal–organic frameworks (Mg–GA MOFs) to enhance alveolar bone regeneration in periodontitis. This hydrogel, made from carboxymethyl chitosan, dextran, and 4-formylphenylboronic acid, allows controlled release of MOFs in response to ROS and pH variations found in the periodontal environment. Laboratory and animal studies confirmed that the hydrogel effectively suppresses inflammation-associated gene and protein expression, thereby supporting bone repair. It also demonstrated potent antibacterial effects against major periodontitis bacteria, *Actinobacillus actinomycetemcomitans* and *P. gingivalis*, as evidenced by live/dead bacterial staining and colony-forming unit assays. Electron microscopy revealed significant structural damage to bacteria treated with MOFs (Fig. 8c). The antibacterial properties resulted from the combined effects of the chitosan-based matrix and the Mg–GA MOFs. Blood compatibility tests showed negligible hemolysis (<2%) for all hydrogel formulations (Fig. 8b), indicating excellent hemocompatibility. Furthermore, the hydrogels promoted the proliferation of RAW 264.7 and MC3T3 cells without altering their typical morphology. Treatment with LPS and MOF-containing hydrogels downregulated iNOS expression (a pro-inflammatory marker), while significantly upregulating anti-inflammatory genes including CD206, IL-10, and TGF-β3. Among these, CSBDX@5MOF showed the most notable effects in enhancing IL-10 and TGF-β3 levels. No significant difference was observed in iNOS expression among hydrogel groups, which remained close to the control (Fig. 8a).

**Fig. 9:**
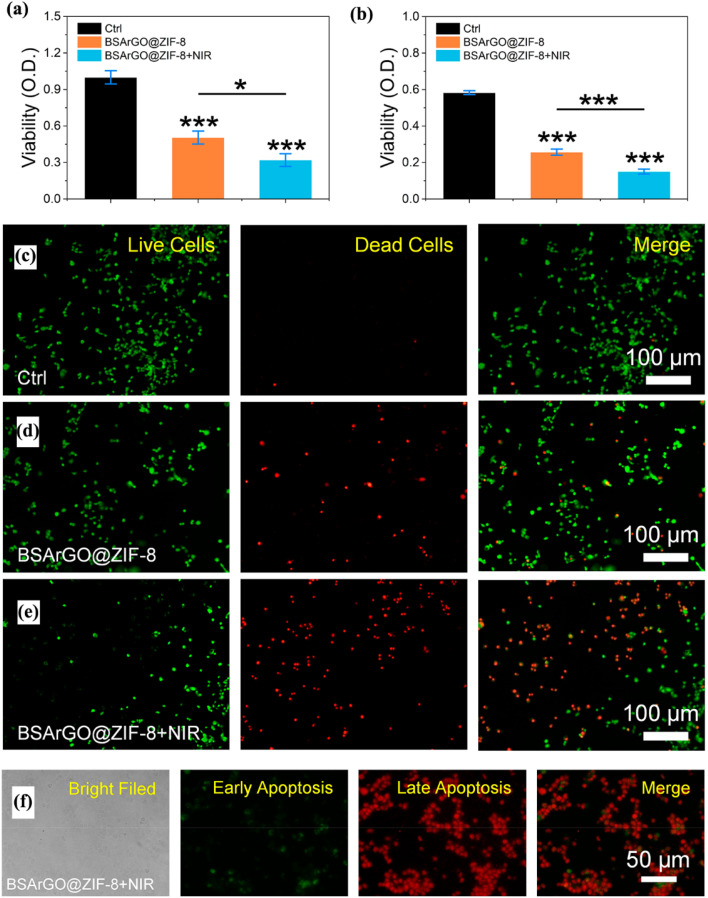
Synergistic effect of photothermal therapy and ion-interference therapy. (a and b) Viability of SCC25 cells and Cal27 cells after treatment with BSArGO@ZIF-8 NSs, with or without NIR irradiation, respectively. (c–e) Live/dead staining of Cal27 cells treated with BSArGO@ZIF-8 NSs, with or without NIR irradiation. (f) Fluorescence detection of early and late apoptosis in Cal27 cells treated with BSArGO@ZIF-8 NSs and NIR irradiation^94^ (reproduced with permission from Lv C., Kang W., Liu S., Yang P., Nishina Y., Ge S., Bianco A. and Ma B., *ACS Nano*, 2022, **16**(7), pp. 11428–11443. Copyright 2022 American Chemical Society).

Lv *et al.*^94^ developed a novel BSArGO@ZIF-8 system consisting of Zn^2+^ and 2-methylimidazole. To enhance the photothermal effect, they reduced graphene oxide (GO) to rGO using ascorbic acid, followed by modifying rGO@ZIF-8 nanosheets with bovine serum albumin (BSA) to create an effective drug delivery carrier. The resulting multifunctional nanoplatform (BSArGO@ZIF-8 NSs) was designed for solid tumor treatment. The *in vivo* antitumor performance of the BSArGO@ZIF-8 NSs was evaluated in BALB/c nude mice with Cal27 xenografts under near-infrared (NIR) irradiation. The results showed that BSArGO@ZIF-8 NSs induced cell apoptosis by triggering Bim-mediated mitochondrial apoptotic pathways, increasing the expression of PUMA/NOXA, and decreasing the levels of Bid/p53AIP1. A total of five injections (BSArGO@ZIF-8 NSs, 4 mg mL^−1^, 50 μL) were administered over 12 days at 48-hour intervals. After this treatment, a significant reduction in tumor size was observed in the BSArGO@ZIF-8 NSs group, with a mass loss ratio of approximately 30%, compared to the saline-treated group. When combined with NIR irradiation, the mass loss ratio of the tumors reached around 70%, further reducing the tumor size (Fig. 9).

**Fig. 10:**
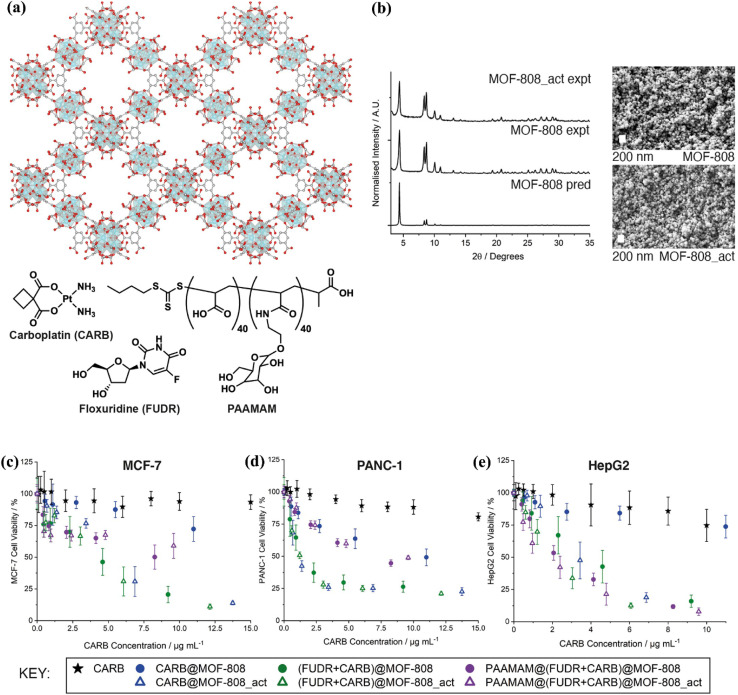
(a) The structural arrangement of MOF-808, highlighting its hexagonal pores, alongside the molecular structures of the chemotherapeutic drugs carboplatin (CARB) and floxuridine (FUDR), as well as the glycopolymer poly(acrylic acid-mannose acrylamide) (PAAMAM). (b) Characterization of MOF-808 and its activated form (MOF-808-act) through XRD analysis, complemented by SEM images of the nanoparticle samples (scale bars: 200 nm). (c–e) Cell viability assessment of MCF-7, PANC-1, and HepG2 cells following 72-hour treatment with CARB-loaded MOFs, compared to treatment with free CARB, measured using the Alamar Blue assay. Viability is presented as a function of CARB concentration, with untreated cells serving as controls. Due to the low loading efficiency of FUDR, comparable graphs based on FUDR concentration could not be generated^95^ (reproduced from Demir Duman F., Monaco A., Foulkes R., Becer C. R. and Forgan R. S., *ACS Appl. Nano Mater.*, 2022, **5**(10), pp. 13862–13873 under a Creative Commons Attribution 4.0 International License).

Demir Duman *et al.*^95^ developed MOF-808, a highly porous material featuring a hexagonal topology. Its structure consists of (Zr_6_O_4_(OH)_4_(CO_2_)_6_·(HCO_2_)_6_) SBUs linked by benzene-1,3,5-tricarboxylic acid (BTC) ligands. The successful synthesis of MOF-808 was confirmed using powder X-ray diffraction and scanning electron microscopy (Fig. 5b). To improve its drug delivery potential, the nanoparticles were coated with a glycopolymer, poly(acrylic acid-mannose acrylamide) (PAAMAM), designed to selectively target cancer cells and enhance chemotherapy effectiveness. The system was tested for the dual drug delivery of floxuridine (FUDR) and carboplatin (CARB), both loaded into the MOF-808 nanoparticles (Fig. 10a). During the drug-loading process, a solvent mixture of methanol (MeOH) and water (H_2_O) was used to dissolve both drugs. Interestingly, this method not only facilitated drug loading but also increased the porosity of some samples, further activating them. A comparative analysis was conducted between the original MOF-808 nanoparticles and their activated counterparts (MOF-808-act), which were treated with the MeOH/H_2_O solution. The study then examined the cytotoxicity and cellular uptake of drug-loaded MOF-808, MOF-808-act, and free PAAMAM in human cancer cell lines MCF-7, PANC-1, and HepG2. After 24 hours, neither the MOF-808 variants nor free PAAMAM showed significant toxicity. However, after 72 hours, the dual-drug-loaded nanoparticles drastically reduced cancer cell viability (Fig. 10c–e). MOF-808 not only improved the therapeutic effects of FUDR and CARB individually but also enhanced their combined cytotoxicity, producing a synergistic effect that increased the potency of the free drugs. These findings highlight the potential of nanoparticle-based drug delivery systems in cancer therapy. The ability of MOF-808 to enhance drug efficacy through combination therapy makes it a promising candidate for future drug delivery applications.

**Fig. 11:**
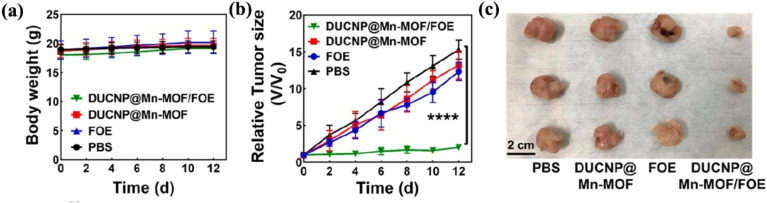
DUCNP@Mn-MOF/FOE exhibits anticancer activity *in vivo*. (a) Body weight variations in mice. (b) DUCNP@Mn-MOF/FOE treatment effectively suppresses tumors *in vivo*. Tumor volumes were determined for each group (*n* = 5, ****p < 0.0001). (c) After the animals were euthanized on day 12, all tumors were isolated and their morphologies studied^96^ (reproduced with permission from Zhao X., He S., Li B., Liu B., Shi Y., Cong W., Gao F., Li J., Wang F., Liu K. and Sheng C., *Nano Lett.*, 2023, **23**(3), pp. 863–871. Copyright 2023 American Chemical Society).

Zhao *et al.*^96^ developed lanthanide-doped upconversion nanoparticles (DUCNPs) capable of converting near-infrared (NIR) light into emissions with shorter wavelengths. These nanoparticles are highly suitable for imaging applications due to their superior photochemical stability, narrow emission spectrum, and significant anti-Stokes shift. Initially, hydrochloric acid was used to eliminate the oleic acid (OA) ligand, enabling surface modification. The nanoparticles were then coated with polyethylene pyrrolidone (PVP), making them water-soluble (DUCNP@PVP). A Mn-MOF layer, consisting of Mn^2+^ and 2,5-dihydroxyterephthalic acid, was subsequently applied *via* a hydrothermal reaction, forming a DUCNP@Mn-MOF core–shell structure. Given its microporous nature and sensitivity to acidic conditions, the Mn-MOF layer rapidly disintegrates in mildly acidic environments, creating additional sites for drug loading. The cytotoxic agent 3-F-10-OH-evodiamine (FOE) was incorporated into the nanoparticles to develop a DDS with potent antitumor properties (DUCNP@Mn-MOF/FOE). The effectiveness of DUCNP@Mn-MOF/FOE was evaluated in 4T1 mice, with intravenous administration of 3 mg kg^−1^ every three days. As illustrated in Fig. 6b and c, this treatment significantly suppressed tumor growth, achieving a 96% tumor growth inhibition (TGI) rate after 12 days. Conversely, treatment groups receiving FOE, DUCNP@Mn–MOF, or PBS exhibited no substantial *in vivo* antitumor effects (Fig. 11).

**Fig. 12:**
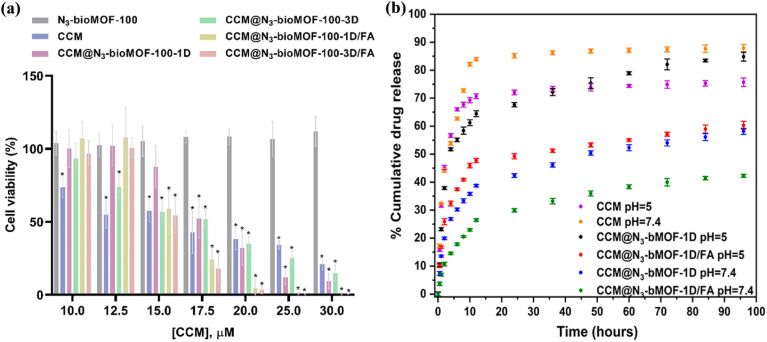
(a) Cytotoxicity effect of free CCM, N_3_-Bio-MOF-100, CCM@N_3_-Bio-MOF-100-1D, CCM@N_3_-Bio-MOF-100-3D, CCM@N_3_-Bio-MOF-100-1D/FA, and CCM@N_3_-Bio-MOF-100-3D/FA against the 4T1 cell line after 48 hours. A two-way ANOVA followed by Tukey post-test, *p < 0.05 when compared with the negative control. (b) Cellular uptake of free CCM, CCM@N_3_-Bio-MOF-100-1D, CCM@N_3_-Bio-MOF-100-3D, CCM@N_3_-Bio-MOF-100-1D/FA, and CCM@N_3_-Bio-MOF-100-3D/FA in the 4T1 cell line. One-way ANOVA followed by Tukey post-test, *p < 0.05 when compared with free CCM^97^ (reproduced with permission from Alves R. C., Schulte Z. M., Luiz M. T., Bento da Silva P., Frem R. C., Rosi N. L., and Chorilli M., *Inorg. Chem.*, 2021, **60**(16), pp. 11739–11744. Copyright 2021 American Chemical Society).

Alves *et al.*^97^ utilized the N_3_-Bio-MOF-100 as a DDS for targeting breast cancer (Fig. 12). The Bio-MOF-100 structure is composed of Zn(ii), adeninate (Ad), and biphenyl dicarboxylate (BPDC), forming an octagonal framework connected by carboxylate bonds. Curcumin (CCM), an anticancer drug, was loaded into this mesoporous material, and to target the system to breast cancer cells, folic acid (FA) was used as a surface linker. The system demonstrated a drug-loading encapsulation efficiency of 25%, with an explosive release of curcumin within the first 48 hours. The cytotoxic effects of this DDS on 4T1 cells were evaluated after 48 hours. The results showed that the highest cytotoxicity was observed with the N_3_-Bio-MOF-100-FA system, where 30 μM of curcumin had the most lethal effect on breast cancer cells. The N_3_-Bio-MOF-100 system alone also exhibited toxic effects on the cells.

In the past decade, bioimaging has emerged as a highly promising tool for achieving rapid, sensitive, and accurate analyses in clinical applications. In response to the growing demand for precise sensing, there has been significant progress in designing and fabricating functional materials. Various organic and inorganic materials, including metallic nanoparticles, graphene oxide (GO), silica nanoparticles, quantum dots, and MOFs, have been explored for the development of biosensors.^98^ The interest in MOFs for biosensing applications has surged due to their diverse structural features and multifunctional properties, which allow for specific molecular recognition and enhance their versatility in various sensing tasks.^99^

In biomedical sensing, MOFs have been widely explored as novel imaging probes for biomedical applications. There are two primary approaches in MOF-based biosensing: (1) MOFs serve as fluorescence quenchers for analytes' fluorophores *via* mechanisms like fluorescence resonance energy transfer (FRET), photoinduced electron transfer (PET), or charge transfer; and (2) MOFs are intentionally designed with fluorescence or luminescence properties that can respond sensitively to their local environment or specific guest molecules. The main focus of research has been on using MOFs as sensors for biomolecules such as DNA, RNA, and enzymes. Additionally, MOFs have been applied to detect various small molecules, including glucose, dopamine, amino acids, and ROS, which are crucial in regulating physiological processes.^97^

**Fig. 13:**
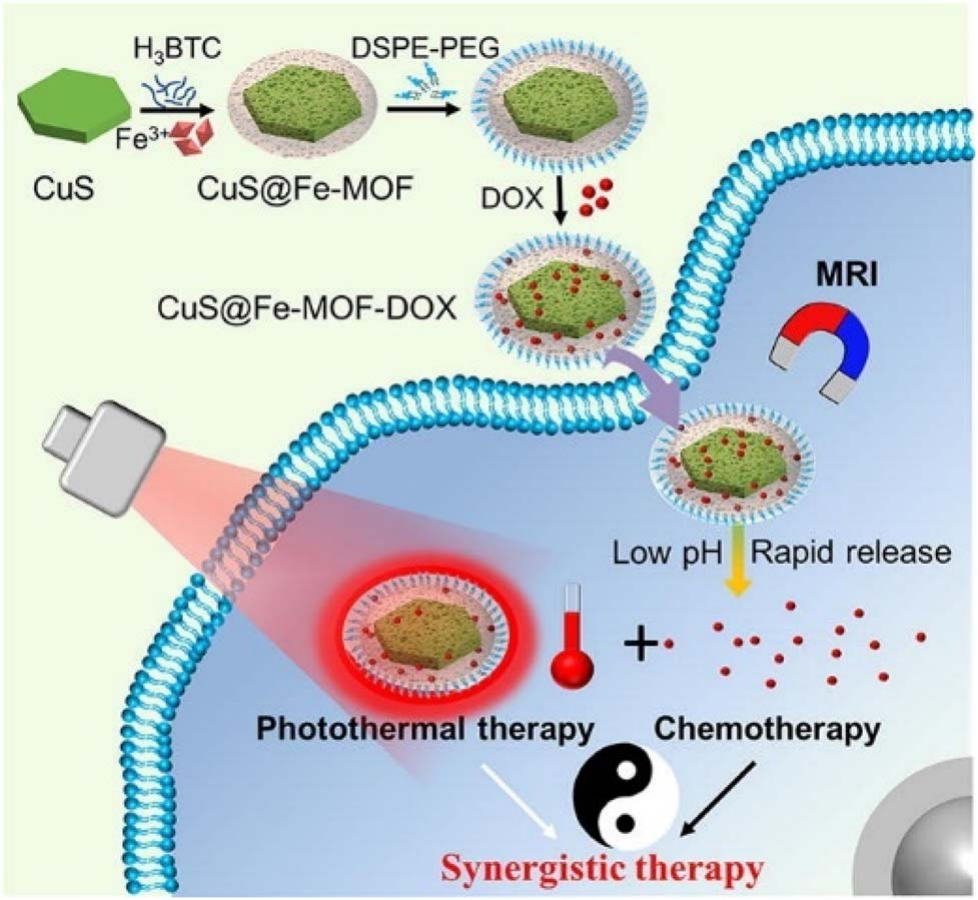
Schematic of CuS@Fe-MOF nanoparticles as a theranostics agent for MRI diagnostic^100^ (reproduced with permission from Wang Z., Yu W., Yu N., Li X., Feng Y., Geng P., Wen M., Li M., Zhang H. and Chen Z., *Chem. Eng. J.*, 2020, **400**, p. 125877. Copyright 2020 Elsevier B.V.).

Wang *et al.*^100^ reported a combination of MOF systems and photothermal agents, such as noble metals or semiconductors, for tumor cell imaging. In this study (Fig. 13), the Fe-MOF system was integrated with CuS, a photothermal agent. CuS nanoparticles, known for their role in destroying cancer cells, absorb strong near-infrared (NIR) light as semiconductor optical agents. The CuS@Fe-MOF nanoparticles were fabricated through the co-growth deposition/assembly method, where an Fe-MOF shell was grown on the surface of CuS nanosheets. These nanoparticles have a hexagonal CuS nanosheet core with an average size of ∼85 nm and an amorphous Fe-MOF shell approximately 16 nm thick. The surface of the CuS@Fe-MOF nanoparticles was modified with lipids to enhance their properties. The nanoparticles exhibited enhanced NIR optical absorption and photothermal conversion efficiency (39.7%), attributed to the localized surface plasmon resonance (LSPR) effect of CuS. Additionally, the Fe-MOF shell provided excellent doxorubicin (DOX) loading capacity (27.5%) and pH-responsive release capabilities. When CuS@Fe-MOF-DOX was injected into tumor-bearing mice, MR and thermal imaging were used to monitor the tumors. The combined photothermal and chemotherapy treatment demonstrated superior tumor inhibition and destruction compared to either photothermal therapy or chemotherapy alone.

**Fig. 14:**
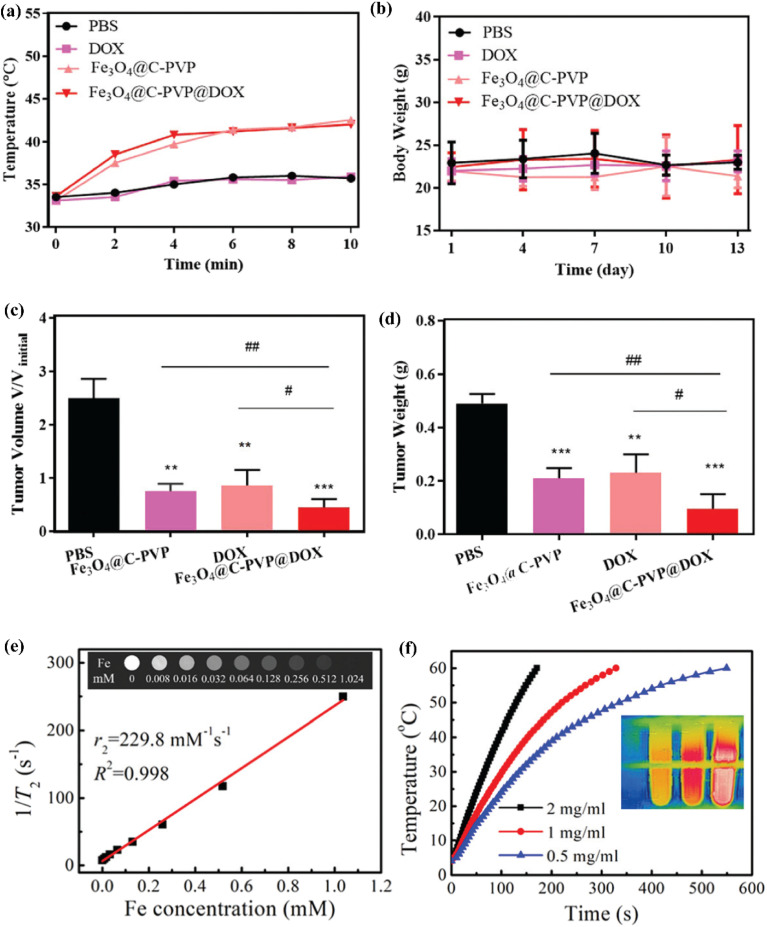
(a and b) Time-dependent variation of tumor surface temperature in mice treated with PBS, DOX (0.23 mg kg^−1^), Fe_3_O_4_@C-PVP (5 mg kg^−1^), and Fe_3_O_4_@C-PVP@DOX (5 mg kg^−1^), along with the corresponding body weight changes during therapy. (c and d) Relative tumor volume progression during the treatment period (*n* = 4) and the final tumor weight at the end of the experiment. (e) *T*_2_ relaxivity profiles of Fe_3_O_4_@C-PVP nanoparticles at different Fe concentrations. The inset presents *in vitro T*_2_-weighted MRI images of Fe_3_O_4_@C-PVP nanoparticles dispersed in 1% agarose with varying Fe concentrations. (f) Temperature evolution of the porous Fe_3_O_4_@C-PVP nanoparticle suspension under an alternating magnetic field of 4.8 kA m^−1^ at 898 kHz (ref. 101) (reproduced from Xiang Z., Qi Y., Lu Y., Hu Z., Wang X., Jia W., Hu J., Ji J. and Lu W., *J. Mater. Chem. B*, 2020, **8**(37), pp. 8671–8683 with permission from the Royal Society of Chemistry).

Xiang *et al.*^101^ detailed the preparation process for Fe-MOFs and their subsequent use in a targeted cancer treatment platform (Fig. 14). Iron(iii) chloride-hexahydrate and 1,4-benzene dicarboxylic acid (H_2_BDC) were dissolved in a DMF solution to synthesize Fe-MOFs. These Fe-MOFs were then pyrolyzed at 500 °C under an argon atmosphere for 10 minutes to obtain Fe_3_O_4_@C nanoparticles. To create Fe_3_O_4_@C-PVP nanoparticles, 0.05 g of Fe_3_O_4_@C nanoparticles and 0.5 g of poly(vinylpyrrolidone) (PVP) were mixed in 50 mL of distilled water and stirred for 24 hours. After centrifugation, the mixture was washed three times with water and ethanol and dried under vacuum overnight. The Fe_3_O_4_@C-PVP nanoparticles were then loaded with a doxorubicin (DOX) solution. Male BALB/c nude mice (4 weeks old) were injected subcutaneously with CAL27 cells (2 × 10^6^ per mouse). Once the tumor volume reached 200–300 mm^3^, the mice were divided into four groups: (1) PBS group; (2) DOX group; (3) Fe_3_O_4_@C-PVP group; (4) Fe_3_O_4_@C-PVP@DOX group. Intratumoral injections were performed on days 1 and 10, with mice being anesthetized and treated with an alternating magnetic field (AMF) for 13 continuous days, with 24-hour intervals between treatments. To evaluate the MRI contrast enhancement effect of the Fe_3_O_4_@C-PVP nanoparticles, *in vitro T*_2_-weighted MRI tests were performed. The results showed that MRI signal intensities decreased as the concentration of Fe_3_O_4_@C-PVP nanoparticles increased, with an *r*_2_ value of 229.8 mM^−1^ s^−1^. This value was lower than that of conventional Fe_3_O_4_ nanoparticles. Additionally, the Fe_3_O_4_@C-PVP@DOX nanoparticles, combined with AMF treatment, demonstrated effective therapeutic outcomes in the oral cancer xenograft model. This system exhibited excellent MRI contrast enhancement, magnetic heating efficiency, DOX loading capacity, and on-demand drug release. Moreover, it showed good biocompatibility and the potential for magnetic-triggered synergistic therapy, combining hyperthermia and chemotherapy, making it a promising candidate for cancer treatment applications.

### 5.2. 3D printing of MOFs for biomedical applications

The 3D printing process operates on the principle of layer-by-layer manufacturing, enabling the direct creation of objects from digital models by sequentially applying layers of material.^108^ In biomedical material development, researchers have successfully employed various 3D printing techniques, including fused deposition modeling (FDM), semi-solid extrusion (SSE), stereolithography (SLA), selective laser sintering (SLS), and inkjet printing. These techniques allow for the fabrication of high-quality, multifunctional materials by incorporating nanomaterials, enhancing the properties of the final product. Nanomaterials incorporated into 3D-printed objects can significantly improve their mechanical, optical, thermal, and electrical properties. However, certain challenges arise due to the inherent brittle nature of photopolymers used in SLA printing. This brittleness makes it difficult to create materials with well-defined mechanical properties. To address this, SLA-printed materials can be strengthened by integrating nanomaterials into the liquid resin. By mixing metal–organic frameworks (MOFs) with the resin, hybrid materials with controlled mechanical properties can be fabricated, making the 3D-printed objects more robust and functional. Additionally, 3D printing serves as an effective platform for enhancing the dispersity, stability, and biocompatibility of nanomaterials. Through the precise control offered by 3D printing, MOFs can be tailored and incorporated into biomedical applications to create materials with improved performance, such as scaffolds for tissue engineering, drug delivery systems, and diagnostic devices.^109,110^ The versatility and precision of 3D printing, combined with the multifunctional properties of MOFs, open up exciting possibilities for advancing biomedical technologies.

**Fig. 15:**
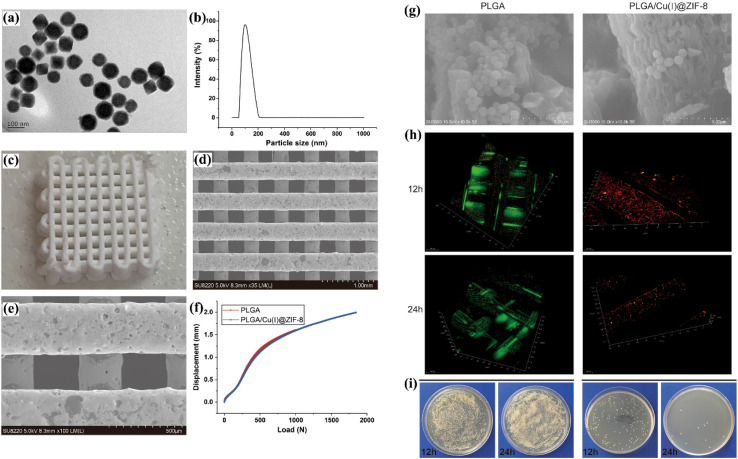
Characterization of Cu(i)@ZIF-8 nanoparticles and PLGA/Cu(i)@ZIF-8 scaffolds: (a) TEM image of Cu(i)@ZIF-8 nanoparticles, (b) particle size distribution of Cu(i)@ZIF-8 nanoparticles, (c) digital image, and (d and e) TEM images of PLGA/Cu(i)@ZIF-8 scaffolds. (f) Load-displacement curve comparing PLGA scaffolds and PLGA/Cu(i)@ZIF-8 scaffolds. (g) Bacterial growth on the surface of both PLGA and PLGA/Cu(i)@ZIF-8 scaffolds. (h) SEM imaging of bacteria on the surface of PLGA and PLGA/Cu(i)@ZIF-8 scaffolds. (i) Live/dead bacterial assessment on the scaffold surfaces after 12 and 24 hours of incubation. (j) Indirect detection of bacterial adhesion on PLGA and PLGA/Cu(i)@ZIF-8 scaffolds using a colony-forming assay^111^ (reproduced from Zou F., Jiang J., Lv F., Xia X. and Ma X., *J. Nanobiotechnol.*, 2020, **18**(1), p. 39 under a Creative Commons Attribution 4.0 International License).

To prepare ZIF-8, Zou *et al.*^111^ dissolved 2-methylimidazole and zinc nitrate hexahydrate (Zn(NO_3_)_2_·6H_2_O) in methanol, followed by sonication of the mixture for 10 minutes, resulting in clear solutions. The resulting product was then recovered by centrifugation, washed three times with methanol, and dried at 60 °C under vacuum overnight. They synthesized Cu(i)@ZIF-8 using a simple adsorption approach, wherein ZIF-8 and cupric chloride were combined in deionized water (Fig. 15a and b). Porous PLGA/Cu(i)@ZIF-8 scaffolds (1 cm in diameter and 0.9 mm in height) were created by combining Cu(i)@ZIF-8 and poly(lactide-*co*-glycolide) (PLGA) through a layer-by-layer 3D printing method. The scaffolds exhibited a porosity of 80.04 ± 5.6% and demonstrated good mechanical properties (Fig. 15c to f). The PLGA/Cu(i)@ZIF-8 scaffolds stimulated the proliferation of murine mesenchymal stem cells (MSCs), enhanced cell adhesion and spreading, and significantly increased MSC osteoblastic differentiation. Moreover, the PLGA/Cu(i)@ZIF-8 scaffolds exhibited remarkable antibacterial properties both *in vitro* and *in vivo* (Fig. 15g to i). Overall, their innovative 3D-printed PLGA/Cu(i)@ZIF-8 scaffolds show significant potential for bone tissue engineering, especially in the treatment of infected bone deformities.

Neus Crespí Sánchez *et al.*^48^ employed microwave-assisted synthesis to rapidly fabricate iron-based MOFs. Their procedure began by dissolving FeCl_3_·6H_2_O in water, followed by the gradual addition of BTC under constant stirring. Before surface modification, MIL-100 MOF was thermally activated at 453 K for 12 hours in a round-bottom flask under a continuous nitrogen atmosphere to produce coordinatively unsaturated sites (CUSs). Functionalization was then achieved by grafting aminomethanesulfonic acid onto the iron sites of the MIL-100 framework, resulting in sulfonated MIL-100-Fe-AMSA. Nitrogen adsorption–desorption isotherms for both MIL-100-Fe and MIL-100-Fe-AMSA revealed characteristics of combined type I and type IV profiles, with notable nitrogen uptake at low relative pressures (*P*/*P*_0_), indicating predominantly microporous structures. The synthesized solids were subsequently filtered, rinsed with ethanol, and air-dried at ambient temperature. For device integration, a 3D-printed column with embedded packing based on a network of interconnected cubes was designed using Rhinoceros 5.0 SR11 32 software. The structure was vertically printed with a support base, consisting of 1016 layers fabricated at a resolution of 0.500 mm using SLA technology. The MIL-100-Fe-AMSA/3D column was created using a simple coating process with concentrated ink. The resulting MIL-100-Fe-AMSA was employed to create a functional device (MIL-100-Fe-AMSA/3D column), which demonstrated high efficiency in the simultaneous extraction and preconcentration of diclofenac (DCF) and ketoprofen. This makes it a promising device for analyzing low levels of emerging pollutants in water.

## 6. Effect of nano/microscale structure of MOF scaffolds on bone regeneration

Biological bone consists of dense and hard connective tissue with remarkable mechanical properties, which are graded in terms of structure and performance. The bone's outer structure, known as the compact bone, is composed of Haversian canals and osteons, while its inner part, the spongy bone, features a trabecular structure with a porosity range of 75–85%. This gradual structural transition from the bone cortex to the spongy regions results in changes in pore distribution and mechanical properties, transitioning from tensile strength to elasticity. Understanding these properties is crucial because the architecture of scaffolds plays a significant role in cellular and molecular behavior. Specifically, scaffold structure influences cell functions at the biological level by either promoting or restricting cellular activities through direct contact. The morphology and size of pores within scaffolds—especially at the nano and microscale—can enhance cell attachment, proliferation, and differentiation, which are critical for effective bone regeneration. The development of MOF scaffolds with tailored nano/microscale architectures offers a promising avenue to mimic the natural bone environment. By adjusting the pore size, surface area, and connectivity of MOF scaffolds, it is possible to create a biomimetic environment that supports cellular processes essential for bone regeneration. Moreover, incorporating bioactive molecules or growth factors into the MOF structure can further promote osteogenesis and accelerate bone healing. Therefore, the design of MOF scaffolds with precise control over their nanoscale and microscale structures is a key factor in improving bone regeneration outcomes.

Several studies have emphasized that the design of bone scaffolds should closely mimic the characteristics of natural bone tissue, particularly its nanotopology, to facilitate successful bone regeneration.^112^ The multiscale structural complexity of scaffold nanostructures plays a pivotal role in regulating cell activity, especially in terms of guiding cellular behavior and enhancing bone tissue formation. At the nanoscale, the scaffold's surface topography can significantly influence the local immune microenvironment, which in turn plays a crucial role in coordinating ossification.^113^ The surface topography of biomaterial scaffolds can directly impact several cellular behaviors, such as cell shape, proliferation, adhesion, and differentiation. Macrophages, which are key mediators of cellular immune responses to biomaterials, are also influenced by the surface topography of the scaffold. Specifically, the topography can modulate macrophage phenotype and activity, promoting pro-inflammatory cytokine release, which is essential for regulating osteogenesis. In the case of calcium-deficient hydroxyapatite, its surface topography has been shown to promote osteogenic cell ossification by stimulating macrophages to release pro-inflammatory cytokines, further enhancing the osteogenic processes.^114^ Moreover, the design of nanostructured materials, such as needle-like hydroxyapatite, has demonstrated its ability to facilitate osteogenic cell ossification by providing a conducive environment for cell adhesion and signaling. This highlights the importance of designing bone scaffolds with specific nanostructures to effectively regulate cellular and immune responses, ultimately enhancing the efficacy of bone regeneration strategies.

On the other hand, scaffolds designed with micropatterns have been shown to significantly influence the alignment of extracellular matrix (ECM) cells and bone tissue. In general, larger pores at the micrometer scale contribute to osteogenesis by promoting the formation of mineralized bone tissue. This is achieved through enhanced blood vessel growth and increased oxygenation, both of which are critical for tissue growth and regeneration. The larger pores allow for better vascularization, which in turn supports the supply of nutrients and oxygen, aiding the development of healthy bone tissue. In contrast, smaller pores at the nanoscale serve a different but equally important function. These nanoscale pores primarily provide more surface area for the adsorption of bioactive molecules, such as growth factors, which are essential for cellular proliferation, differentiation, and matrix formation. Additionally, nanoscale pores enhance the transport of nutrients and the removal of metabolic wastes, ensuring that cells remain in a favorable environment for growth and activity. This balance between micro and nanoscale porosity is crucial for optimizing scaffold design to promote both osteogenesis and cell viability, ultimately improving the outcomes of bone regeneration therapies.^113^

Biomimetic 3D biomaterial nano/micro scaffolds provide suitable microenvironments for skeletal regeneration by closely mimicking the natural structure and properties of bone. Among these, nano- or micro-scaffolds based on MOFs are gaining significant attention due to their unique properties. The nano-topography of MOF-based scaffolds can modulate osteoblast lineage cell activity directly, enhancing bone differentiation and creating a favorable microenvironment for bone tissue regeneration.^115^ The microstructural patterns in these scaffolds are designed to promote osteoblastic proliferation, differentiation, mineralization, and overall bone formation. These scaffolds provide an ideal platform for the attachment of osteoblastic lineage cells, enabling them to form new bone tissue. Additionally, the expression of bone-related genes can be regulated through chemical signals, facilitating more efficient bone formation. In recent years, multifunctional scaffolds that incorporate nanoparticles or bioactive molecules have become increasingly popular. These advanced scaffolds serve as tools to stimulate the proliferation and differentiation of osteoblasts, which are key players in bone regeneration. By loading nanoparticles or molecules that support osteogenesis, these scaffolds enhance the regenerative capacity and therapeutic potential of bone tissue engineering.^116^

## 7. Combination of MOFs with nanocomposites for enhancing bone regeneration

As research into bone healing and treatment progresses, an increasing number of studies are focusing on the integration of MOFs into bone repair and therapeutic applications.^117^ When combined with other materials, particularly nanomaterials, these MOFs form what is known as MOF-nanocomposites. These composites often exhibit enhanced physicochemical properties and functionalities, offering advantages over the original MOFs.^118^ The primary method of characterizing MOF-nanocomposites involves identifying the modifying agent used or the specific application intended for the nanocomposite.^119^ MOF-based nanocomposites can be broadly categorized into four main types: bio/MOF, which are designed to interact effectively with biological systems; metal/MOF, which incorporate metal components to enhance mechanical or biological performance; non-metal/MOF, where non-metal elements are used to modify properties; and semiconductor/MOF, which are utilized in applications such as photothermal therapy or photocatalysis.^120^ Each type offers unique benefits for bone regeneration and repair, contributing to the optimization of bone tissue engineering strategies.

**Fig. 16:**
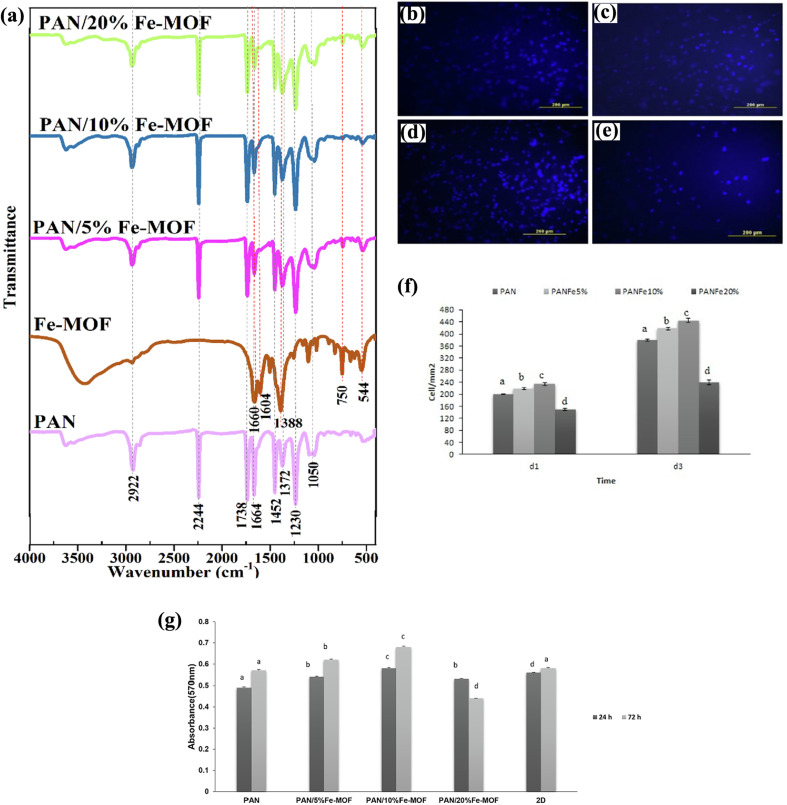
(a) FT-IR spectra of Fe-MOF, PAN, and as-prepared PAN/*x*% Fe-MOF nanocomposites. (b–f) Fluorescent microscopic observation of HUVEC cells cultured on scaffolds for 3 days. The cells are stained with DAPI, and cell density within the section samples is shown. (g) MTT assay at 24 h and 72 h time points^122^ (reproduced with permission from Ramezani M. R., Ansari-Asl Z., Hoveizi E. and Kiasat A. R., *Mater. Chem. Phys.*, 2019, **229**, pp. 242–250. Copyright 2019 Elsevier B.V.).

Moris *et al.*^121^ discovered that by combining MOF-801 with a gelatin matrix, they were able to create a promising nanocomposite scaffold for bone tissue engineering and regeneration. In this research, they synthesized a zirconium-based metal–organic framework, MOF-801, and incorporated it into the gelatin matrix to produce the nanocomposite bone scaffold using the freeze-drying method. The results showed improved mechanical and biological properties, including enhanced compressive strength (15 ± 0.05 MPa) and apatite formation in simulated body fluid. The sustained release of zirconium ions and fumarate promoted mineralization and osteoblastic activity. The biocompatibility of the scaffold was confirmed through MTT and crystal violet assays, while Alizarin red and ALP activity assays demonstrated increased calcium mineralization in MG-63 cells. In other research, Ramezani *et al.*^122^ explored the characteristics of new nanofibrous polymeric/MOF scaffolds for applications in medicine, particularly in drug delivery, tissue engineering, and wound healing. They produced three types of nanocomposites by combining polyacrylonitrile (PAN) with Fe(iii) metal–organic framework (Fe-MOF) using a method called electrospinning. Techniques such as SEM, TEM, and FTIR confirmed that the Fe-MOF had been successfully incorporated into the polymer matrix, forming fibrous structures. Biological assessments revealed that PAN/5% Fe-MOF and PAN/10% Fe-MOF scaffolds exhibited superior cell viability, proliferation, and biocompatibility compared to pure PAN and PAN/20% Fe-MOF. These scaffolds promoted cell attachment and growth, showed no signs of inflammatory response in living organisms, and demonstrated good stability (Fig. 16).

The similarity of hydroxyapatite (HA) to natural bone on a chemical level has prompted extensive research into utilizing synthetic HA as a substitute for bone and as a replacement in biomedical applications. Nanofibrous composites consisting of bioactive HA have been regarded as a favorable material for regenerating bone.^123^ Sarkar *et al.*^124^ developed a 3D nanocomposite of cellulose-hydroxyapatite loaded with a drug-containing metal–organic framework (HA/DMOF) for bone tissue engineering. The study demonstrated that the dexamethasone-loaded metal–organic framework (DMOF) nanoparticles (60–80 nm) were successfully integrated into the HA/cellulose nanocomposite. The HA/DMOF nanocomposite exhibited mechanical properties (compressive strength and modulus) similar to cancellous bone. It also provided a sustained release of dexamethasone over 4 weeks, which was longer than the release from DMOF particles alone. Furthermore, the HA/DMOF nanocomposite was compatible with pre-osteoblast cells and enhanced their alkaline phosphatase activity and mineralization. In conclusion, the HA/DMOF nanocomposite shows potential as a drug delivery system for orthopedic applications to facilitate bone regeneration.

Li *et al.*^125^ conducted a study on the impact of ZIF-8@AHT, a modified alkali and heat-treated titanium framework, on osteogenesis. Their findings indicated that ZIF-8@AHT enhances cell bioactivity, boosts extracellular matrix mineralization, increases collagen and osteoprotegerin (OPG) secretion, and upregulates osteogenic genes and proteins. OPG is known for its role in inhibiting osteoclast differentiation. Moreover, the nanoparticles demonstrate strong osteogenic properties at the bone-implant interface. Their porous structure also enables the loading of osteogenic substances. ZIF-8 additionally exhibits angiogenic, antibacterial, and hemostatic properties, which promote bone healing. MOFs like ZIF-8 are increasingly being recognized for their multifunctional capabilities and their potential applications in bioengineering, bone tissue engineering, and bone disease treatment. Furthermore, ZIF-8 activates Mitogen-Activated Protein Kinase (MAPK) signaling pathways in bone repair and promotes osteogenesis in rat bone marrow stem cells (rBMSCs) by entering cells through specialized transport mechanisms.

The researchers have found that incorporating MOFs into biocompatible nanocomposites has significant potential for bone tissue engineering and regeneration. These MOF-nanocomposites have demonstrated improved mechanical, biological, and therapeutic properties compared to individual materials. The combined advantages of MOFs with the nanocomposite matrix emphasize the potential of this composite system for regenerative orthopedic therapies. The findings suggest that these nanocomposites warrant further investigation as a multifunctional platform for bone repair and regeneration (Table 4).

**Table 4:** Comparing the advantages and disadvantages of different types of MOFs in bone tissue

Type of MOF	Advantages	Disadvantages	Ref.
ZIF-8	(1) Development of extracellular matrix mineralization	(1) Further studies needed on ZIF-8, its composites, and applications	111 and 133–135
	(2) Upregulation of osteogenesis-related genes (Alp and Runx2)	(2) Toxicity	
	(3) Promotion of ALP activity		
	(4) Inhibition of *Streptococcus mutans* growth		
	(5) Recognized as beneficial coating materials for implant surface modification		
	(6) Potential use in biodegradable scaffolds and drug delivery systems		
UiO-66	(1) Induction of bone regeneration	(1) Because zirconium (Zr) has a higher atomic number than calcium, UiO-66 appears opaque on X-ray images than natural bone. As a result, it may interfere with the ability to observe new bone growth in the affected areas	136
	(2) No cytotoxicity		
	(3) Promoted new bone formation and collagen		
Mil-88a	(1) Mil-88a could be easily synthesized and has high biocompatibility	(1) Significant cytotoxicity at concentrations >10 μg mL^−1^ (reduced cell viability)	137
	(2) Mil-88a could significantly promote the expression of OA anabolism-related genes, such as Col2, and also significantly inhibit the expression of OA catabolism-related genes, such as MMP13	(2) The exact mechanism of ROS scavenging remains unclear	
		(3) Some limitations in hydrogen storage capacity and need for improvement	
Cu-MOF-74	(1) Antibacterial activity	(1) Biocompatibility assessments revealed enhanced cell proliferation at Cu-MOF-74 concentrations ≤0.2%, while concentrations ≥0.5% induced cytotoxicity	138
	(2) Osteogenic differentiation enhancement		
	(3) Biocompatibility at optimal concentrations		
Cu-HKUST-1	Promising antimicrobial activity and revealed biocompatibility toward human dermal fibroblasts up to a concentration of 1000 μg mL^−1^	—	139
MIL-53	(1) Enhanced adsorption of extracellular matrix (ECM) proteins such as laminin, fibronectin, and perlecan, which improves cell adhesion	(1) The interaction between cells and the scaffold needs further in-depth studies	140
	(2) Improved bioactivity and biocompatibility of the titanium scaffold		
	(3) Increased stiffness of endothelial cells, promoting activation of endothelial tip cells and angiogenesis		
	(4) Facilitated rapid and sufficient vascularization in bone regeneration		
	(5) The “breathing” property of MIL-53(Fe) enables better interaction between the scaffold and vascular endothelial cells		
Mg-MOF	(1) High osteoconductivity	(1) The cement is only suitable for non-load-bearing bone defects, which limits its use in high-stress areas	141
	(2) Enhanced mechanical strength		
	(3) Strong antibacterial properties		
	(4) Anti-inflammatory effects		
	(5) Improved osteogenic differentiation		
	(6) Suitable for non-load-bearing bone defects		
Fe-MOF	(1) Inhibition of TfR2	—	142
	(2) ROS scavenging ability		
	(3) Activation of the BMP pathway		
	(4) Enhanced osteogenic gene and protein expression (*in vitro*)		

Designing and synthesizing suitable MOFs for tissue engineering and bone regeneration involves considering multiple factors, such as the structure and properties of the damaged tissue, utilizing safe manufacturing techniques, and selecting appropriate metallic ions, ligands, and functional groups for tissue regeneration.^126^ For example, when using NMOFs as drug carriers, modifying the pore size can change the loading capacity, which can be adjusted by changing the multimodal organic ligands.^127,128^ In addition, MOFs possess properties that allow them to respond to stimuli, enabling the precise discharge of therapeutic substances like drugs in NMOFs, growth factors, or other bioactive molecules. This facilitates improved bone healing through different types of stimulation, including pH, temperature, magnetic fields, humidity, light, redox reactions, pressure, and ions, all of which are highly advantageous in tissue engineering. pH-responsive NMOFs among various MOF-based stimuli-responsive systems have garnered significant interest in regenerative medicine due to their ability to release drugs, genes, small molecules, and ions in specific environments, particularly in tissues infected with bacteria that have acidic conditions.^129^ Zheng *et al.*^130^ reported a study on a multifunctional nano platform (IL4-MOF@CaP) inspired by embryonic ossification for bone regeneration. The platform features a magnesium-gallate framework and a biodegradable calcium phosphate shell that enables the controlled release of bioactive factors in response to pH changes. This promotes a favorable healing environment by supporting the resolution of inflammation and enhancing angiogenesis. When combined with collagen scaffolds, the platform significantly improves *in vivo* bone regeneration, creating distinct bone island patterns. Overall, this approach shows promise in mimicking natural developmental processes to enhance tissue regeneration in bone defect repair.

**Fig. 17:**
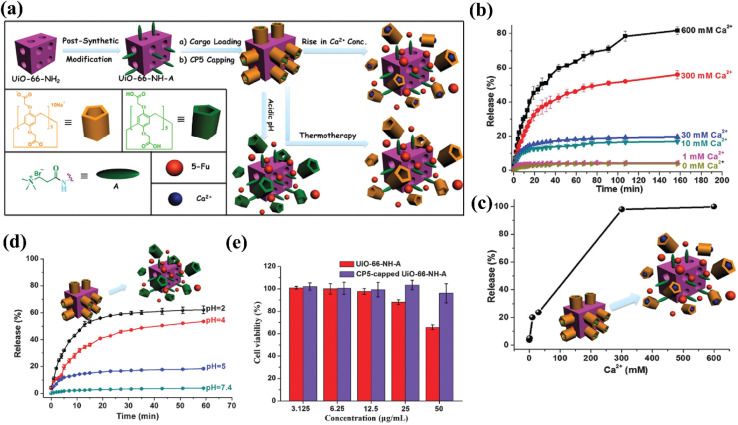
(a) Schematic representation of stimuli-responsive mechanized Zr-MOFs (UiO-66-NH_2_). (b–d) Controlled release profiles of 5-Fu-loaded, CP5-capped UiO-66-NH_2_-A. (e) MTT cytotoxicity assay of 293 cells treated with UiO-66-NH_2_-A and CP5-capped UiO-66-NH_2_-A at various concentrations^131^ (reproduced from Tan L. L., Song N., Zhang S. X. A., Li H., Wang B. and Yang Y. W., *J. Mater. Chem. B*, 2016, **4**(1), pp. 135–140 with permission from the Royal Society of Chemistry).

Tan *et al.*^131^ stated that bone tumor tissues have a lower pH (more acidic) than normal blood and tissues (pH 7.4). This is because the bone tumor cells have lower lysosomal pH levels than healthy human cells (Fig. 17). These cells can directly break down the bone matrix and release a high concentration of Ca^2+^ ions. The high concentration of Ca^2+^ is challenging to measure at the moment of release. The acidic extracellular environment around the osteoclasts (bone-resorbing cells) and bone tumor cells leads to osteolysis and bone breakdown. This increased bone resorption further contributes to the release of Ca^2+^ from the bones. Additionally, bone tumors produce parathyroid hormone, prostaglandin E, and vitamin D sterols, stimulating additional bone resorption. The release of Ca^2+^ from the bones due to direct breakdown by tumor cells and increased osteolysis/resorption results in a transfer of calcium from the bone fluid to the blood, leading to hypercalcemia, characterized by abnormally high calcium levels in the blood.

In response to this, the researchers proposed developing mechanized Zr-based MOFs (Zr-MOFs) with multi-stimuli responsive supramolecular gates that can release drugs (5-fluorouracil) in a controlled manner in the bone tumor environment, triggered by the acidic pH and high Ca^2+^ concentrations, and further triggered by hyperthermia.^131^ Therefore, the control of Ca^2+^ and pH can also reduce negative effects and additionally support the healing of bones within a living organism. Even though there have been limited studies of materials that respond to changes in ionic concentration, it is important to highlight that levels of electrolytes can be a significant indicator for a variety of illnesses. As a result, combining this approach with advancements in material science could lead to more promising methods for precise bone therapy and bone regeneration.^132^

## 8. Conclusion

The development of biomimetic nano- and microstructured scaffolds based on MOFs has emerged as a promising approach for bone regeneration. These scaffolds provide a controlled microenvironment that enhances osteoblastic differentiation, osteoimmune responses, and overall bone tissue repair. The integration of bioactive functionalization strategies has further expanded their potential, enabling simultaneous osteogenesis stimulation, angiogenesis, and targeted imaging, thus advancing theranostic scaffold applications. By mimicking the hierarchical structure of natural bone, MOF-based scaffolds offer enhanced mechanical stability, biocompatibility, and drug-loading efficiency. Their tunable porosity and surface properties allow precise control over drug release and cellular interactions, making them highly effective for bone tissue engineering. Additionally, the incorporation of bioactive molecules, growth factors, and nanoparticles into MOFs has further improved their regenerative potential, accelerating bone healing.

Despite these advancements, several challenges remain. The cytotoxicity of metal ions, the long-term stability of MOFs in physiological environments, and their degradation mechanisms require further investigation. Additionally, scalability, reproducibility, and regulatory considerations must be addressed to facilitate clinical translation. Future research should focus on optimizing synthesis techniques, employing green fabrication approaches, and refining surface modifications to enhance the safety and effectiveness of MOF-based scaffolds.

Future studies should investigate the use of alternative, less cytotoxic metal ions or organic linkers to avoid potential toxicity concerns. In-depth *in vivo* studies are also needed to understand the long-term behavior and biodegradation profiles of these scaffolds in various animal models. Moreover, the development of 3D-printed or patient-specific MOF-based scaffolds could enhance clinical applicability by allowing customization to patient-specific anatomical and biological requirements. In addition, exploring synergistic combinations of MOFs with other biocompatible polymers or ceramics could open new avenues for multifunctional composite scaffolds with superior regenerative capacity. Ultimately, the interdisciplinary collaboration between materials scientists, bioengineers, and medical researchers will be crucial in bridging the gap between laboratory research and clinical applications, paving the way for the next generation of personalized and multifunctional biomaterials in regenerative medicine.

## Abbreviations

3D: Three-Dimensional
AMF: Alternating Magnetic Field
BSA: Bovine Serum Albumin
BTC: Benzene-1,3,5-Tricarboxylic Acid
CaP: Calcium Phosphate
CARB: Carboplatin
CCM: Curcumin
COFs: Covalent Organic Frameworks
CPs: Coordination Polymers
CUSs: Coordinatively Unsaturated Sites
DDS: Drug Delivery System
DMF: Dimethylformamide
DOX: Doxorubicin
DUCNPs: Lanthanide-Doped Upconversion Nanoparticles
ECM: Extracellular Matrix
FA: Folic Acid
Fe-MOF: Iron-Based Metal–Organic Framework
FRET: Fluorescence Resonance Energy Transfer
FUDR: Floxuridine
GO: Graphene Oxide
HA: Hydroxyapatite
ILAG: Ion and Liquid-Assisted Grinding
IRMOFs: Isoreticular MOFs
LAG: Liquid-Assisted Grinding
LSPR: Localized Surface Plasmon Resonance
MAPK: Mitogen-Activated Protein Kinase
MeOH: Methanol
MG-63: Human Osteosarcoma Cell Line
MIL: Materials Institute Lavoisier
MOFs: Metal–Organic Frameworks
MOF-808: Zirconium-Based MOF
MTX: Methotrexate
NIR: Near-Infrared
NMOFs: Nanostructured Metal–Organic Frameworks
OA: Oleic Acid
OPG: Osteoprotegerin
PBS: Phosphate-Buffered Saline
PCN: Porous Coordination Network
PCPs: Porous coordination polymers
PET: Photoinduced Electron Transfer
PVP: Polyvinylpyrrolidone
rGO: Reduced Graphene Oxide
ROS: Reactive Oxygen Species
SLA: Stereolithography
SSA: Specific Surface Area
SUBs: Secondary Building Units
TBAP: Tetra-*n*-Butylammonium Perchlorate
TGI: Tumor Growth Inhibition
TUNEL: Terminal Deoxynucleotidyl Transferase dUTP Nick End Labeling
UiO-66: University of Oslo-66 Metal–Organic Framework
ZIFs: Zeolitic Imidazolate Frameworks
Zr-MOFs: Zirconium-Based Metal–Organic Frameworks

## Conflicts of interest

The authors declare that the research was conducted in the absence of any commercial or financial relationships that could be construed as a potential conflict of interest.

## Data Availability

The data presented in this study are available on request from the corresponding author.
